# *Ligusticum chuanxiong* CO_2_ Oil as a Natural Antimicrobial Agent for Postharvest Preservation of Fresh White Radish: *In Vitro*, *In Situ* and Storage Evaluation

**DOI:** 10.3390/foods15162936

**Published:** 2026-08-21

**Authors:** Miroslava Kačániová, Yunke Yu, Minhang Qiao, Zhaojun Ban, Li Li, Chen Cunkun, Stefania Garzoli, Alessandro Bianchi

**Affiliations:** 1 Institute of Horticulture, Faculty of Horticulture and Landscape Engineering, Slovak University of Agriculture, Trieda Andreja Hlinku 2, 94976 Nitra, Slovakia; aerfabuer@163.com (Y.Y.); qomohoo@163.com (M.Q.); 2 School of Medical & Health Sciences, VIZJA University, Okopowa 59, 01043 Warszawa, Poland; 3 Zhejiang Provincial Collaborative Innovation Center of Agricultural Biological Resources Biochemical Manufacturing, School of Biological and Chemical Engineering, Zhejiang University of Science and Technology, Hangzhou 310023, China; banzhaojun@zust.edu.cn; 4 Key Laboratory for Agro-Products Postharvest Handling of Ministry of Agriculture and Rural Affairs, College of Biosystems Engineering and Food Science, Zhejiang University, Hangzhou 310058, China; lili1984@zju.edu.cn; 5 Institute of Agricultural Products Preservation and Processing Technology, Tianjin Academy of Agricultural Sciences (National Engineering and Technology Research Center for Preservation of Agricultural Products (Tianjin)), Tianjin 300384, China; cck0318@126.com; 6 Department of Chemistry and Technologies of Drug, Sapienza University, P. le Aldo Moro, 5, 00185 Rome, Italy; stefania.garzoli@uniroma1.it; 7 Department of Agriculture, Food and Environment, University of Pisa, Via del Borghetto 80, 56124 Pisa, Italy

**Keywords:** *Ligusticum chuanxiong*, CO_2_ extract, cis-ligustilide, antimicrobial activity, vapour-phase antimicrobial activity, insecticidal activity, MALDI-TOF MS, food preservation, microbiota, vacuum packaging

## Abstract

The present study investigated the chemical composition, antimicrobial, insecticidal, and food preservation potential of *Ligusticum chuanxiong* CO_2_ oil (LCCO) standardised to a ligustilide content of ≥35%. The volatile composition of LCCO was characterised by GC-MS and found to be dominated by cis-ligustilide (63.3%), senkyunolide (15.8%), 3-butylidenephthalide (5.4%), and trans-neocnidilide (4.9%). *In vitro* antimicrobial activity was evaluated by disc diffusion and broth microdilution against six bacterial and five yeast reference strains. The largest inhibition zones were observed against *Enterococcus faecalis* (14.33 mm) and *Staphylococcus aureus* (13.67 mm). MIC_50_ values ranged from 0.064 mg/mL (*Yersinia enterocolitica*) to 3.492 mg/mL (*Candida tropicalis*), while MIC_90_ values ranged from 0.151 to 3.800 mg/mL. *In situ* vapour-phase antimicrobial activity was evaluated on pear, banana, white radish, and carrot at concentrations of 62.5–500 µL/L, demonstrating a clear concentration-dependent inhibitory effect across all tested food matrices and microorganisms. Insecticidal activity against *Callosobruchus maculatus* and *Megabruchidius dorsalis* was confirmed under fumigation conditions, with LC_50_ values of 17.50% and 20.99% and LC90 values of 83.10% and 87.60%, respectively. The effect of LCCO, applied alone and in combination with vacuum packaging, on the microbiological quality of fresh white radish was evaluated over 28 days of refrigerated storage at 4 °C. The combined treatment resulted in the lowest total viable count (2.75 log CFU/g) and coliform bacteria count (2.58 log CFU/g) on day 28. A total of 404 bacterial isolates were identified using MALDI-TOF MS Biotyper, comprising 22 species. *Acinetobacter pittii* was the predominant species (33%), followed by *Pantoea agglomerans* (7%) and *Serratia nematodiphila* (7%). No *Pseudomonas* species were recovered from the EO vacuum treatment. Overall, these findings demonstrate the potential of LCCO, particularly in combination with vacuum packaging, as a natural antimicrobial agent for the postharvest preservation of fresh white radish.

## 1. Introduction

Fresh vegetables represent a critical component of the global food supply, yet postharvest losses attributable to microbial spoilage remain a persistent challenge [1]. The Food and Agriculture Organization of the United Nations estimated in 2024 that approximately 20–40% of fresh produce is lost annually due to phytopathogen attack and spoilage microorganisms during storage and distribution [2]. These losses carry significant economic, environmental, and food security implications, particularly in the context of a growing global population. White radish (*Raphanus sativus* L.) is a root vegetable widely cultivated and consumed across Europe and Asia, valued for its nutritional composition, including glucosinolates, vitamin C, and dietary fibre, as well as its culinary versatility. Despite its commercial importance, information regarding the species-level composition of the spoilage microbiota associated with fresh white radish during refrigerated storage remains limited.

The postharvest protection of fresh vegetables has historically relied on synthetic chemical preservatives and fungicides, including benzoates, sorbates, imazalil, and thiabendazole, often combined with modified atmosphere packaging and refrigeration [3,4]. However, consumer behaviour is undergoing a measurable shift: according to recent market research, nearly one in two consumers globally purchased more fresh and unprocessed foods over the past year, and approximately 30% are actively reducing their intake of products containing synthetic additives and artificial preservatives [5]. This trend, combined with increasing regulatory scrutiny of synthetic preservative residues on fresh produce, has substantially accelerated interest in natural, clean-label antimicrobial alternatives [6].

Plant-derived volatile extracts, including essential oils and supercritical CO_2_ extracts obtained from aromatic and medicinal plants, have emerged as one of the most intensively investigated categories of natural bioactive substances with potential application in food preservation. Their broad-spectrum antimicrobial activity and compatibility with active packaging and vapour-phase delivery systems make them particularly attractive for postharvest applications on fresh produce [3,6]. Among the most relevant delivery strategies is *in situ* vapour-phase application, in which volatile antimicrobial compounds are released into the headspace of sealed packages, inhibiting surface microbial growth without direct contact with the food matrix [7]. This approach is especially well-suited to root vegetables and leafy produce, where direct application may affect sensory properties at higher concentrations.

*Ligusticum chuanxiong* Hort. (Umbelliferae), known in traditional Chinese medicine (TCM) as Chuanxiong, is a perennial herbaceous plant cultivated primarily in Sichuan Province, China, with a well-documented history of medicinal use spanning nearly two millennia. The dried rhizome is widely used in TCM for promoting blood circulation, alleviating pain, and treating gynaecological and cardiovascular conditions [8]. Beyond its medicinal role, *L. chuanxiong* is also consumed as a food: the tender leaves are used in salads and stir-fried dishes, and the rhizome is incorporated into soups, teas, and medicinal wines, reflecting its dual status as both a medicinal herb and a food ingredient in East Asian culinary traditions [9]. The volatile CO_2_ extract of the rhizome, commonly referred to as Chuanxiong oil, is characterised by a phthalide-rich composition, with ligustilide, senkyunolide, and butylidenephthalide among the dominant constituents, together with smaller amounts of terpenoids and other volatile compounds [10,11].

The bioactivity of *Ligusticum chuanxiong* CO_2_ oil (LCCO) has been documented across a range of pharmacological studies, demonstrating sedative, analgesic, neuroprotective, anti-inflammatory, and vasodilatory effects [11,12]. In the context of plant pathology, Cui et al. [10] demonstrated significant antifungal activity of Chuanxiong oil against *Sclerotium rolfsii*, identifying butylidenephthalide as one of the principal active constituents; the oil exhibited an EC_50_ value of 81.79 mg/L and provided effective in vivo control of peanut stem rot. Similarly, Chen et al. [13] reported antifungal activity of *L. chuanxiong*-derived extracts against several phytopathogenic fungi, with ligustilide and senkyunolide A contributing substantially to the observed effects. However, despite this documented biological activity, the antibacterial potential of LCCO against food-associated pathogens and spoilage microorganisms, as well as its application as a natural preservative for fresh vegetables, remain largely unexplored.

The present study employs a commercial *Ligusticum chuanxiong* CO_2_ oil (LCCO) standardised to a ligustilide content of ≥35%. The product is characterised by a phthalide-rich composition, with ligustilide serving as the principal marker compound. The use of a standardised CO_2_-derived extract facilitates reproducibility, enables comparison among studies, and supports the development of future quality-controlled applications of *L. chuanxiong*-derived bioactive products.

MALDI-TOF MS Biotyper (Bruker Daltonics) has become established as a reference method for rapid culture-based identification of food-associated microorganisms at the species level, offering speed and reliability beyond those achievable by conventional biochemical methods. Its application to the microbiota of fresh vegetables has generated valuable information on spoilage community composition and dynamics; however, comprehensive MALDI-TOF MS-based characterisation of the spoilage microbiota associated with fresh white radish during refrigerated storage has not been previously reported.

The present study therefore aimed to: (i) characterise the chemical composition of LCCO by GC-MS; (ii) evaluate its *in vitro* antimicrobial activity against a panel of reference bacterial and yeast strains using disc diffusion and microdilution assays, including MIC_50_ and MIC_90_ determination by probit analysis; (iii) assess its *in situ* vapour-phase antimicrobial activity on pear, banana, white radish, and carrot model systems; (iv) evaluate its insecticidal activity against *Callosobruchus maculatus* and *Megabruchidius dorsalis*; and (v) determine the effect of LCCO application, with and without vacuum packaging, on the microbiological quality and microbiota composition of fresh white radish during 28 days of refrigerated storage at 4 °C using total viable count, coliform bacteria enumeration, and MALDI-TOF MS Biotyper identification. We hypothesised that the phthalide-rich composition of LCCO would exert inhibitory activity against dominant spoilage microorganisms associated with white radish and consequently improve its microbiological stability during refrigerated storage. Although the antimicrobial properties of *Ligusticum chuanxiong* CO_2_ oil have been reported in traditional medicine and pharmacological research, its potential for postharvest preservation of fresh vegetables has not yet been investigated. Therefore, the present study evaluated the antimicrobial, vapour phase antimicrobial, and food preservation potential of LCCO in fresh white radish, while simultaneously providing species-level characterization of the natural spoilage microbiota using MALDI-TOF MS Biotyper.

## 2. Materials and Methods

### 2.1. Characterization of Chuanxiong Rhizome CO_2_ Oil (LCCO)

Chuanxiong rhizome CO_2_ oil obtained from the rhizome of *Ligusticum chuanxiong* Hort. by supercritical carbon dioxide extraction was used as the tested bioactive substance in the present study. The oil was supplied by Guilin Four Seasons Sunshine (Guilin, Guangxi, China; batch number 20241106). According to the manufacturer’s specification, the product was characterised as a red to brownish-red oily liquid with the characteristic aroma of *L. chuanxiong*. The declared quality parameters included a moisture content ≤ 0.5%, a ligustilide content ≥ 35%, a heavy metal content < 10 ppm, arsenic < 0.5 ppm, lead < 1 ppm, cadmium < 1 ppm, and aflatoxin < 0.2 ppb. Prior to analysis and biological testing, the oil was stored in a dark glass container at 4 °C and protected from direct sunlight.

### 2.2. Chemical Composition of LCCO

The volatile composition of *Ligusticum chuanxiong* CO_2_ oil (LCCO) was analyzed by gas chromatography–mass spectrometry (GC–MS). Analyses were performed using a Clarus 500 instrument (PerkinElmer, Waltham, MA, USA) equipped with a flame ionization detector (FID) and coupled to a mass spectrometer. Separation of the compounds was achieved on a Varian Factor Four VF-5 capillary column (60 m × 0.32 mm i.d., film thickness 1.0 µm). The oven temperature programme was set as follows: an initial temperature of 60 °C, increased at a rate of 6 °C/min to 220 °C, where it was maintained for 20 min. Helium was used as the carrier gas at a constant flow rate of 1 mL/min [14].

Compound identification was performed by comparing the obtained mass spectra with those contained in the Wiley 2.2 and NIST 11 mass spectral libraries. Linear retention indices (LRI) were calculated using a homologous series of *n*-alkanes (C_8_–C_25_) analysed under identical chromatographic conditions and subsequently compared with published literature values. The relative abundance of individual constituents was expressed as the percentage peak area of the total chromatographic area. No internal standard or response correction factor was applied. All analyses were performed in triplicate.

### 2.3. Evaluation of Antimicrobial Activity

#### 2.3.1. Tested Microorganisms and Culture Conditions

The antimicrobial activity of *Ligusticum chuanxiong* CO_2_ oil (LCCO) was evaluated against a panel of reference bacterial and yeast strains. The bacterial panel comprised the Gram-positive species *Enterococcus faecalis* (CCM 4224), *Listeria monocytogenes* (CCM 4699), and *Staphylococcus aureus* subsp. *aureus* (CCM 4223), as well as the Gram-negative species *Escherichia coli* (CCM 3954), *Salmonella enterica* subsp. *enterica* (CCM 3807), and *Yersinia enterocolitica* (CCM 7204^T^). The yeast panel consisted of *Candida albicans* (CCM 8186), *Candida glabrata* (CCM 8270), *Candida krusei* (CCM 8271), *Candida parapsilosis* (CCM 8260), and *Candida tropicalis* (CCM 8223).

Bacterial strains were cultivated in Mueller–Hinton broth (MHB; Oxoid, Basingstoke, UK), whereas yeasts were cultivated in Sabouraud dextrose broth (SDB; Oxoid, Basingstoke, UK). Bacterial cultures were incubated at 37 °C for 24 h, while yeast cultures were incubated at 25 °C for 24 h. Prior to antimicrobial testing, all inocula were adjusted to the turbidity of a 0.5 McFarland standard, corresponding to approximately 1.5 × 10^8^ CFU/mL.

#### 2.3.2. Disc Diffusion Assay

The antimicrobial activity of *Ligusticum chuanxiong* CO_2_ oil (LCCO) was initially evaluated using the disc diffusion assay. Sterile filter paper discs (6 mm diameter; Oxoid, Basingstoke, UK) were impregnated with 10 μL of LCCO and placed onto the surface of Mueller–Hinton agar (MHA; Oxoid, Basingstoke, UK) plates inoculated with bacterial suspensions or Sabouraud dextrose agar (SDA; Oxoid, Basingstoke, UK) plates inoculated with yeast suspensions. Following incubation for 24 h at 37 °C for bacteria and 25 °C for yeasts, the diameters of inhibition zones were measured in millimetres. Discs containing only the solvent served as negative controls. Commercial antimicrobial discs were used as positive controls: cefoxitin (30 μg/disc) for Gram-positive bacteria, gentamicin (10 μg/disc) for Gram-negative bacteria, and fluconazole (25 μg/disc) for yeasts (Oxoid, Basingstoke, UK). All experiments were performed in triplicate [15].

#### 2.3.3. Determination of MIC_50_ and MIC_90_ by the Microdilution Method

The minimum inhibitory concentrations (MIC_50_ and MIC_90_) of LCCO were determined using the broth microdilution method in sterile 96-well microtiter plates. Each well contained 50 μL of microbial suspension adjusted to the 0.5 McFarland standard and 50 μL of a two-fold serial dilution of LCCO prepared in Mueller–Hinton broth (MHB) for bacteria or Sabouraud dextrose broth (SDB) for yeasts. The oil was dispersed directly in the culture medium during preparation of the dilution series without the addition of organic solvents. Tested concentrations ranged from 10 mg/mL to 0.00485 mg/mL.

Negative control wells contained only sterile culture medium, whereas growth control wells contained inoculated culture medium without LCCO. Following incubation for 24 h at 37 °C for bacteria and 25 °C for yeasts, microbial growth was quantified spectrophotometrically by measuring absorbance at 570 nm using a GloMax^®^ microplate reader (Promega, Madison, WI, USA). MIC_50_ and MIC_90_ values were calculated by probit analysis based on the measured growth inhibition relative to the untreated growth control. All experiments were performed in triplicate [16].

### 2.4. In Situ Evaluation of the Antimicrobial Activity of LCCO on Food Model Matrices

The *in situ* antimicrobial activity of *Ligusticum chuanxiong* CO_2_ oil (LCCO) was evaluated on selected plant-derived food model matrices, including pear (*Pyrus communis* L.), banana (*Musa acuminata* Colla), white radish (*Raphanus sativus* L.), and carrot (*Daucus carota* L.). Fresh plant materials were washed, cut into slices of approximately 0.5 cm thickness, and allowed to dry under sterile conditions. Individual slices were aseptically transferred into sterile 60-mm Petri dishes and inoculated with standardized bacterial or yeast suspensions.

LCCO was applied in the vapour phase using sterile filter paper discs attached to the inner surface of the Petri dish lids. The discs were impregnated with LCCO solutions prepared in ethyl acetate to achieve final concentrations of 500, 250, 125, and 62.5 μL/L of headspace. Control samples contained filter paper discs treated with ethyl acetate only. The dishes were hermetically sealed with Parafilm^®^ and incubated for 7 days at 37 °C for bacterial strains and 25 °C for yeasts.

Following incubation, antimicrobial activity was evaluated by digital image analysis using ImageJ software version 1.54k (National Institutes of Health, Bethesda, MD, USA). The extent of microbial growth inhibition was determined semi-quantitatively based on the relative area of microbial growth on the inoculated food surfaces according to a modified procedure described by Kačániová et al. [17]. Results were expressed as microbial growth inhibition (MGI, %) relative to the untreated control and calculated according to the following equation:

MGI (%) = [(C − T)/C] × 100(1)

where C represents the area of microbial growth in the untreated control and T represents the area of microbial growth in the treated sample [16].

### 2.5. Storage Experiment with White Radish

Fresh white radish (*Raphanus sativus* L.) with a total weight of approximately 2.5 kg was purchased from a local retail market on the day the experiment was initiated. After removal of visible impurities and the outer peel, the radishes were rinsed with distilled water and allowed to drain on a sterile stainless-steel rack at laboratory temperature. Mechanical drying was not applied to avoid tissue damage. Subsequently, the radishes were sliced into pieces of approximately 0.5 cm thickness and aseptically divided into portions weighing 25 g.

The experiment was designed to evaluate the effects of vacuum packaging and the application of *Ligusticum chuanxiong* CO_2_ oil (LCCO) on the microbiological quality of white radish during refrigerated storage. Five experimental groups were established:

- C1 (−V/−LCCO/−Tween) = non-vacuum packaged samples without LCCO or Tween 80 (negative control);
- C2 (+V/−LCCO/−Tween) = vacuum-packaged samples without LCCO;
- C3 (−V/+Tween/−LCCO) = non-vacuum packaged samples treated with Tween 80 solution only (emulsifier control);
- C4 (−V/+LCCO/+Tween) = non-vacuum packaged samples treated with LCCO;
- EO (+V/+LCCO/+Tween) = vacuum-packaged samples treated with LCCO.

For the treated groups (C4 and EO), LCCO was applied as a 1% (*w*/*w*) emulsion prepared in 0.5% Tween 80 solution. In group C3, only 0.5% Tween 80 solution was applied. The treatment solutions were evenly distributed over the surface of each 25 g radish portion under aseptic conditions. Samples were gently mixed for approximately 1 min to ensure homogeneous distribution while minimizing mechanical damage to the plant tissue.

Samples assigned to groups C2 and EO were vacuum packaged using a vacuum packaging machine VA-0010 (Concept, Choceň, Czech Republic) and food-grade vacuum bags. Vacuum packaging was employed to reduce oxygen availability within the package and thereby limit the growth of aerobic spoilage microorganisms. Samples from groups C1, C3, and C4 were placed into sterile polyethylene resealable bags without vacuum application.

All samples were stored at 4 ± 1 °C for 28 days. Microbiological analyses were performed on day 0 using three untreated baseline samples collected prior to treatment application and subsequently on days 1, 7, 14, 21 and 28 of storage. At each post-treatment sampling point, three independent replicates from each of the five experimental groups were analysed, resulting in 15 samples per sampling day (5 groups × 3 replicates). Consequently, a total of 78 samples were included in the storage experiment.

### 2.6. Microbiological Evaluation of White Radish

At each sampling time, 25 g of white radish (*Raphanus sativus* L.) was aseptically transferred into a sterile stomacher bag containing 225 mL of sterile peptone water (Oxoid, Basingstoke, UK), corresponding to a 1:10 dilution. Samples were homogenized for 2 min using a Stomacher homogenizer. Subsequently, ten-fold serial dilutions were prepared in sterile peptone water, and 100 μL aliquots from appropriate dilutions were surface-plated onto selective culture media.

Total viable counts (TVC) were determined on Plate Count Agar (PCA; Oxoid, Basingstoke, UK) following incubation at 30 °C for 48–72 h in accordance with ISO 4833-1:2013 [18]. Coliform bacteria were enumerated on Violet Red Bile Lactose Agar (VRBL; Oxoid, Basingstoke, UK) after incubation at 37 °C for 24–48 h according to ISO 4832:2006 [19]. Microbial counts were expressed as log CFU/g of fresh sample. Microscopic fungi and yeasts were enumerated on Dichloran Rose Bengal Chloramphenicol Agar (DRBC; Oxoid, Basingstoke, UK) after incubation at 25 °C for 5 days in accordance with ISO 21527-1:2008 [20]. The presence of *Salmonella* spp. was determined on Xylose Lysine Deoxycholate Agar (XLD; Oxoid, Basingstoke, UK) after incubation at 37 °C for 24–48 h according to ISO 6579-1:2017 [21]. Microbial counts were expressed as log CFU/g of fresh sample.

### 2.7. Identification of Microorganisms by MALDI-TOF MS

Representative colonies exhibiting distinct morphological characteristics were selected from the culture media and subcultured to obtain pure isolates. Identification was performed using a MALDI-TOF MS Biotyper system (Bruker Daltonics, Bremen, Germany). Protein extraction was carried out according to the ethanol–formic acid extraction protocol recommended by the manufacturer.

Briefly, a single colony was suspended in 300 μL of distilled water and centrifuged at 10,000× *g* for 2 min. Following removal of the supernatant, 900 μL of absolute ethanol was added and the suspension was centrifuged again under the same conditions. The resulting pellet was air-dried and subsequently resuspended in 30 μL of 70% formic acid and 30 μL of acetonitrile. One microlitre of the extract was spotted onto a polished steel MALDI target plate and allowed to dry at room temperature. Subsequently, 1 μL of HCCA matrix solution (α-cyano-4-hydroxycinnamic acid) was applied to each spot.

Mass spectra were acquired and analysed using the MALDI Biotyper software package version 3.1 and compared with the reference database provided by the manufacturer. Identification scores were interpreted according to the Bruker Daltonics criteria as follows: scores ≥ 2.000 indicated reliable species-level identification, scores between 1.700 and 1.999 indicated probable genus-level identification, and scores < 1.700 were considered unreliable.

### 2.8. Evaluation of Insecticidal Activity

The insecticidal activity of *Ligusticum chuanxiong* CO_2_ oil (LCCO) was evaluated against two storage pest species, *Callosobruchus maculatus* (Fabricius, 1775) and *Megabruchidius dorsalis* (Fahraeus, 1839) (Coleoptera: Chrysomelidae). Groups of 50 adult individuals of each species were transferred into sterile Petri dishes. Sterile filter paper discs placed inside the dishes were treated with 100 μL of LCCO at concentrations of 100, 50, 25, 12.5, 6.25, and 3.125%. To ensure uniform dispersion, the oil was diluted in 0.1% polysorbate 80 (Tween 80; Sigma-Aldrich, St. Louis, MO, USA).

The Petri dishes were hermetically sealed with Parafilm^®^ to minimise the loss of volatile compounds and incubated at 25 ± 2 °C for 24 h. This experimental setup enabled insect exposure to the volatile constituents of LCCO under fumigation conditions. Control groups consisted of filter paper discs treated only with 0.1% Tween 80 solution without LCCO. Following the exposure period, the numbers of live and dead insects were recorded, and mortality percentages were calculated. All experiments were performed in triplicate.

The lethal concentration values (LC_50_ and LC_90_) were estimated by probit analysis based on the concentration–mortality relationship obtained from the fumigation bioassay. LC_50_ and LC_90_ were defined as the concentrations causing 50% and 90% mortality of the tested insect population, respectively.

### 2.9. Statistical Analysis

Statistical processing and figure generation were carried out using JMP Student Edition 18.2 (SAS Institute, Cary, NC, USA). Results are reported as mean values accompanied by their standard deviation (SD), with each analysis performed in triplicate. Before conducting the inferential analyses, compliance with ANOVA assumptions was confirmed: residual normality was examined through the Shapiro–Wilk test, while variance homogeneity was verified using Bartlett’s test. Since these assumptions were satisfied, a one-way ANOVA was employed to evaluate differences among treatments. When the model indicated significant effects (*p* ≤ 0.05), Tukey’s HSD procedure was applied for post hoc comparisons.

For the storage experiment, a two-way ANOVA was performed separately for total viable counts and coliform counts, with treatment and storage time as fixed factors. The treatment × storage time interaction was included in the model. When significant effects were detected (*p* ≤ 0.05), Tukey’s HSD test was applied for post hoc multiple comparisons. For the comparisons presented different letters within each storage day indicate significant differences among treatments according to Tukey’s HSD test.

Probit analysis was applied for the estimation of MIC_50_, MIC_90_, LC_50_, and LC_90_ values. For the storage experiment, the effects of treatment and storage time on microbial counts were evaluated using ANOVA, with differences considered statistically significant at *p* ≤ 0.05.

## 3. Results

### 3.1. Chemical Composition of LCCO

The chemical composition of LCCO is presented in Table 1. A total of 21 compounds were identified, accounting for 99.9% of the total chromatographic area. The oil was characterised by a phthalide-rich composition, with cis-ligustilide as the dominant constituent (63.3%), followed by senkyunolide (15.8%), 3-butylidenephthalide (5.4%), trans-neocnidilide (4.9%), and 3-butylphthalide (3.5%). Among the remaining identified compounds, trans-ligustilide (2.2%) and β-eudesmene (1.5%) were present at lower concentrations, while 2-methoxy-4-vinylphenol (0.9%), spathulenol (0.5%), α-cubebene (0.4%), and *p*-cymene (0.2%) were detected at low or trace levels. Monoterpene hydrocarbons, including α-pinene, β-pinene, α-terpinene, limonene, γ-terpinene, and terpinolene, were detected at concentrations below 0.3%, while terpinene-4-ol was also present at a similarly low level. α-Pinene and β-pinene were detected only in trace amounts.

### 3.2. Disc Diffusion Assay

The antimicrobial activity of LCCO determined by the disc diffusion assay is presented in Table 2. Among Gram-positive bacteria, the largest inhibition zone was observed against *E. faecalis* (14.33 mm), followed by *S. aureus* (13.67 mm) and *L. monocytogenes* (13.33 mm). Among Gram-negative bacteria, *E. coli* exhibited an inhibition zone of 11.67 mm, while *Y. enterocolitica* and *S. enterica* showed inhibition zones of 10.33 mm and 9.67 mm, respectively. Among the tested yeasts, inhibition zones ranged from 7.67 mm for *C. albicans* and *C. parapsilosis* to 8.33 mm for *C. glabrata*, *C. krusei*, and *C. tropicalis*. The antibiotic controls produced substantially larger inhibition zones against all tested microorganisms, ranging from 28.33 mm (*C. tropicalis*) to 32.67 mm (*S. enterica*).

### 3.3. Minimum Inhibitory Concentrations (MIC_50_ and MIC_90_)

The MIC_50_ and MIC_90_ values of LCCO determined by the broth microdilution method and probit analysis are presented in Table 3. Among Gram-negative bacteria, *Y. enterocolitica* was the most susceptible microorganism, with an MIC_50_ of 0.064 mg/mL and an MIC_90_ of 0.151 mg/mL. *E. coli* and *S. enterica* showed higher MIC_50_ values of 1.848 mg/mL and 2.270 mg/mL, with corresponding MIC_90_ values of 1.977 mg/mL and 2.773 mg/mL, respectively. Among Gram-positive bacteria, *S. aureus* was the most susceptible strain, with an MIC_50_ of 0.288 mg/mL and an MIC_90_ of 1.198 mg/mL, followed by *E. faecalis* (MIC_50_ = 1.342 mg/mL, MIC_90_ = 1.803 mg/mL) and *L. monocytogenes* (MIC_50_ = 1.763 mg/mL, MIC_90_ = 2.000 mg/mL). Among the tested yeasts, *C. glabrata* exhibited the lowest MIC_50_ (0.575 mg/mL) and MIC_90_ (0.929 mg/mL), whereas *C. tropicalis* was the least susceptible, with an MIC_50_ of 3.492 mg/mL and an MIC_90_ of 3.800 mg/mL. *C. krusei* showed an MIC_50_ of 2.732 mg/mL and an MIC_90_ of 2.970 mg/mL, while *C. albicans* and *C. parapsilosis* exhibited intermediate values.

### 3.4. In Situ Antimicrobial Activity of LCCO

#### 3.4.1. *In Situ* Antimicrobial Activity of LCCO on Pear

**Figure 1:**
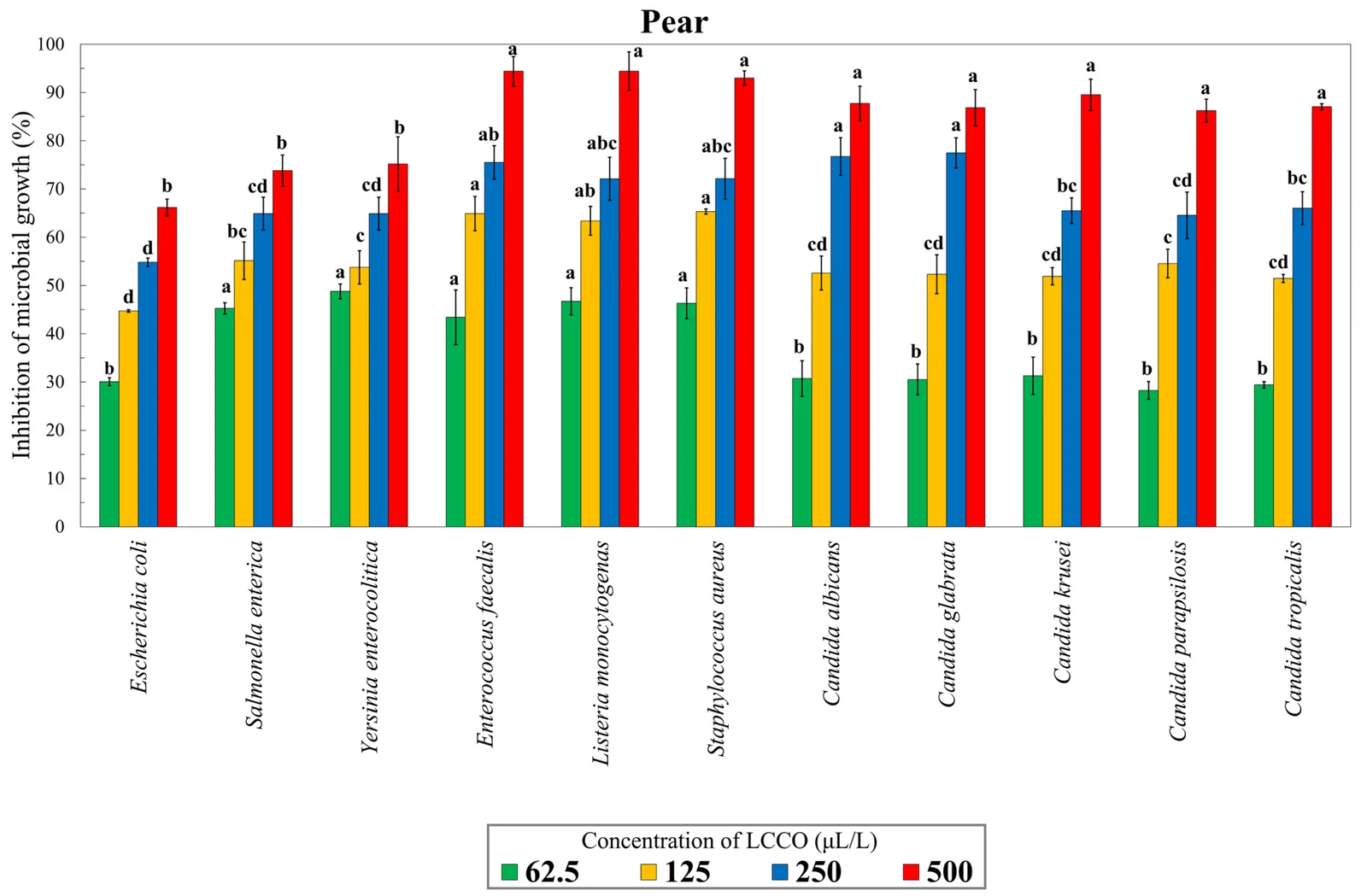
*In situ* analysis of the inhibition of microbial growth (%) by the vapor phase of LCCO at different concentrations (μL/L) in pear. Data are the mean (bars indicate ± standard deviation) of three replicates. Different letters (a–d) indicate statistically significant differences within each EO concentration (*p* ≤ 0.05).

The *in situ* antimicrobial activity of LCCO on pear is presented in Figure 1. At the highest tested concentration (500 µL/L), LCCO exhibited strong inhibitory activity against all tested microorganisms. The highest MGI values were recorded for *L. monocytogenes* (98.92%), followed by *E. faecalis* (94.40%) and *S. aureus* (92.98%). Among Gram-negative bacteria, *Y. enterocolitica*, *S. enterica*, and *E. coli* exhibited MGI values of 75.18%, 73.80%, and 66.17%, respectively. Among the tested yeasts, *C. krusei* showed the highest inhibition (89.53%), followed by *C. albicans* (87.75%), *C. tropicalis* (87.05%), *C. glabrata* (86.83%), and *C. parapsilosis* (86.26%).

At 250 µL/L, the MGI values decreased for all tested microorganisms, ranging from 54.82% (*E. coli*) to 76.13% (*L. monocytogenes*). At 125 µL/L, the inhibitory activity further declined, with MGI values ranging from 44.72% (*E. coli*) to 65.32% (*S. aureus*). At the lowest tested concentration (62.5 µL/L), MGI values ranged from 29.42% (*C. tropicalis*) to 48.77% (*Y. enterocolitica*), confirming a concentration-dependent reduction in antimicrobial activity against all tested microorganisms.

#### 3.4.2. *In Situ* Antimicrobial Activity of LCCO on Banana

**Figure 2:**
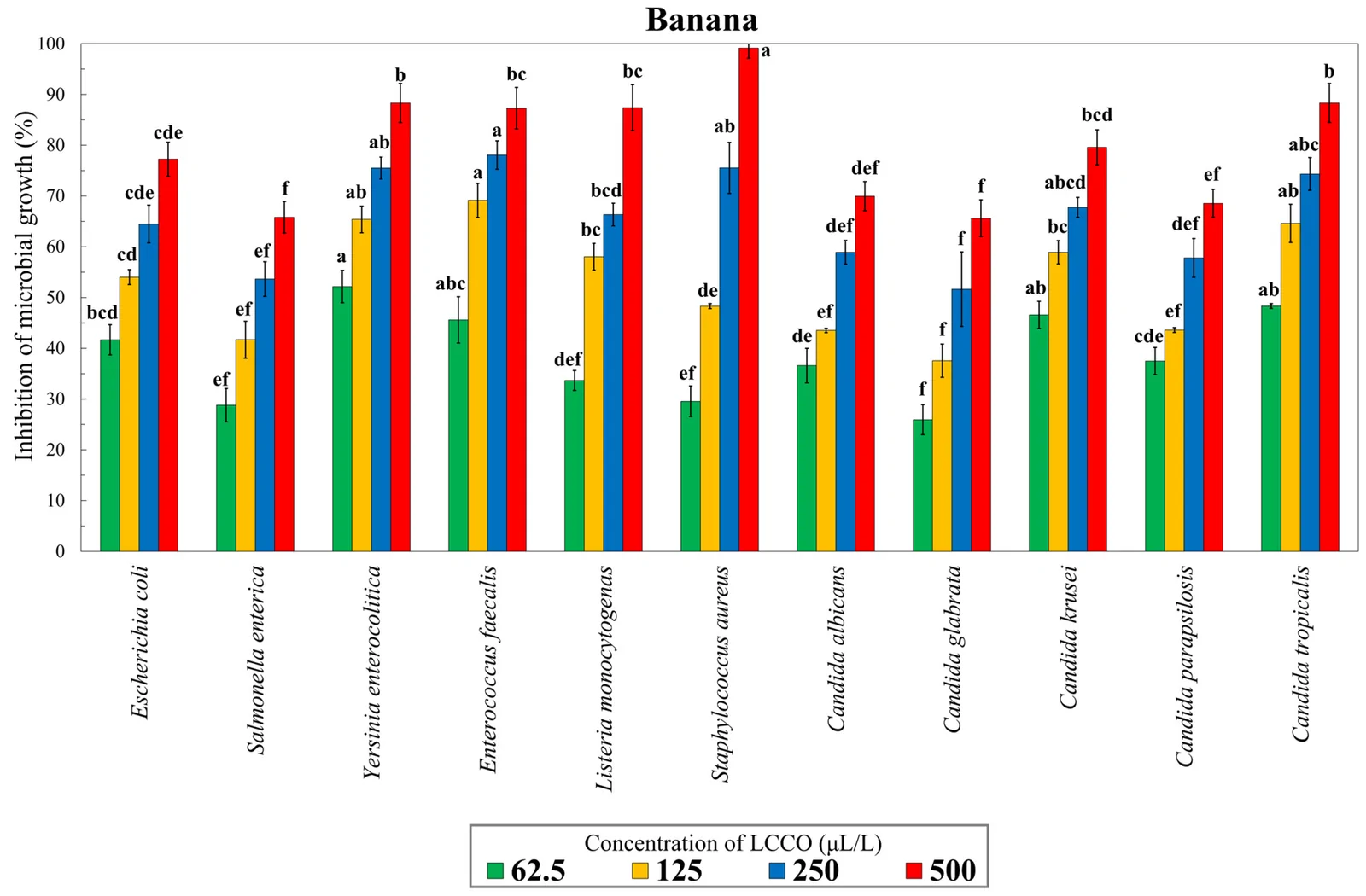
*In situ* analysis of the inhibition of microbial growth (%) by the vapor phase of LCCO at different concentrations (μL/L) in banana. Data are the mean (bars indicate ± standard deviation) of three replicates. Different letters (a–f) indicate statistically significant differences within each EO concentration (*p* ≤ 0.05).

The *in situ* antimicrobial activity of LCCO on banana is presented in Figure 2. At the highest tested concentration (500 µL/L), LCCO exhibited strong inhibitory activity against all tested microorganisms. The highest MGI value was recorded for *S. aureus* (99.11%), followed by *C. tropicalis* (88.31%), *Y. enterocolitica* (88.31%), *L. monocytogenes* (87.40%), *E. faecalis* (87.30%), and *E. coli* (77.25%). *S. enterica* exhibited an MGI of 65.80%. Among the tested yeasts, *C. krusei* showed the highest inhibition (79.57%), followed by *C. albicans* (69.96%), *C. parapsilosis* (68.55%), and *C. glabrata* (65.63%).

At 250 µL/L, the MGI values decreased for all tested microorganisms, ranging from 51.66% (*C. glabrata*) to 75.53% (*S. aureus*). At 125 µL/L, the inhibitory activity further declined, with MGI values ranging from 37.57% (*C. glabrata*) to 65.38% (*Y. enterocolitica*). At the lowest tested concentration (62.5 µL/L), MGI values ranged from 25.94% (*C. glabrata*) to 52.16% (*Y. enterocolitica*), confirming a concentration-dependent reduction in antimicrobial activity against all tested microorganisms.

#### 3.4.3. *In Situ* Antimicrobial Activity of LCCO on White Radish

**Figure 3:**
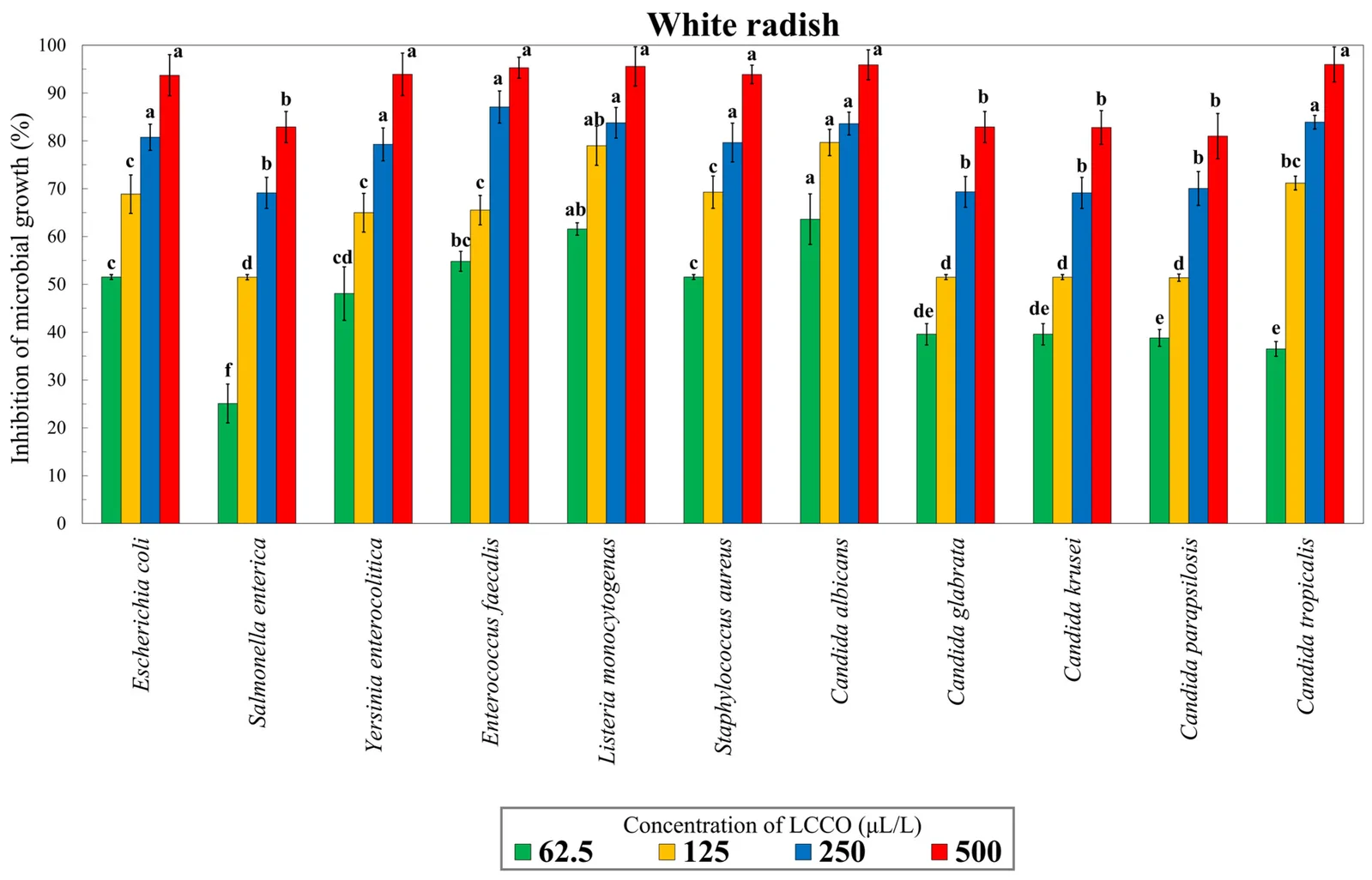
*In situ* analysis of the inhibition of microbial growth (%) by the vapor phase of LCCO at different concentrations (μL/L) in white radish. Data are the mean (bars indicate ± standard deviation) of three replicates. Different letters (a–f) indicate statistically significant differences within each EO concentration (*p* ≤ 0.05).

The *in situ* antimicrobial activity of LCCO on white radish is presented in Figure 3. At the highest tested concentration (500 µL/L), LCCO exhibited the strongest inhibitory activity among all tested food matrices. Among Gram-positive bacteria, *L. monocytogenes* (95.58%), *E. faecalis* (95.30%), and *S. aureus* (93.89%) showed the highest MGI values. Among Gram-negative bacteria, *Y. enterocolitica* (93.92%) and *E. coli* (93.72%) exhibited similarly high inhibition, whereas *S. enterica* was less susceptible, with an MGI of 82.90%. Among the tested yeasts, *C. tropicalis* (95.97%) and *C. albicans* (95.91%) showed the highest inhibition, while *C. glabrata* (82.90%), *C. krusei* (82.81%), and *C. parapsilosis* (80.99%) exhibited moderate inhibitory activity.

At 250 µL/L, the MGI values decreased for all tested microorganisms, ranging from 66.02% (*C. glabrata*) to 90.80% (*E. faecalis*). At 125 µL/L, the inhibitory activity further declined, with MGI values ranging from 51.01% (*C. parapsilosis*) to 82.71% (*L. monocytogenes*). At the lowest tested concentration (62.5 µL/L), MGI values ranged from 35.08% (*C. tropicalis*) to 61.56% (*L. monocytogenes*), confirming a concentration-dependent reduction in antimicrobial activity against all tested microorganisms.

#### 3.4.4. *In Situ* Antimicrobial Activity of LCCO on Carrot

**Figure 4:**
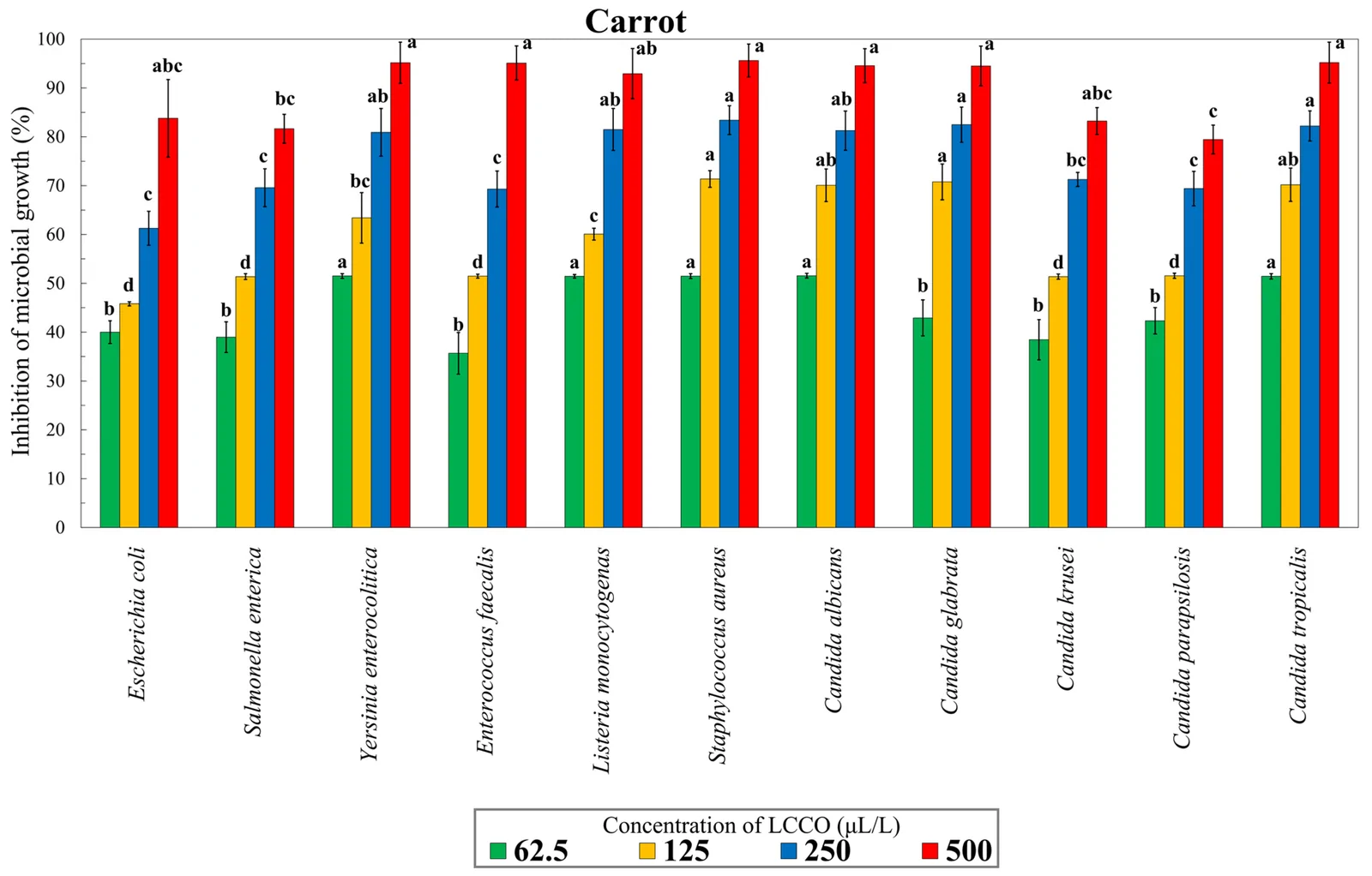
*In situ* analysis of the inhibition of microbial growth (%) by the vapor phase of LCCO at different concentrations (μL/L) in carrot. Data are the mean (bars indicate ± standard deviation) of three replicates. Different letters (a–d) indicate statistically significant differences within each EO concentration (*p* ≤ 0.05).

The *in situ* antimicrobial activity of LCCO on carrot is presented in Figure 4. At the highest tested concentration (500 µL/L), LCCO exhibited strong inhibitory activity against all tested microorganisms. Among Gram-positive bacteria, *S. aureus* (95.61%), *E. faecalis* (95.11%), and *L. monocytogenes* (92.92%) showed the highest MGI values. Among Gram-negative bacteria, *Y. enterocolitica* exhibited the highest inhibition (95.18%), followed by *E. coli* (83.79%) and *S. enterica* (81.66%). Among the tested yeasts, *C. tropicalis* (95.20%), *C. albicans* (94.57%), and *C. glabrata* (94.49%) showed the highest inhibition, whereas *C. krusei* (83.24%) and *C. parapsilosis* (79.45%) exhibited moderate inhibitory activity.

At 250 µL/L, the MGI values decreased for all tested microorganisms, ranging from 61.27% (*C. parapsilosis*) to 86.20% (*L. monocytogenes*). At 125 µL/L, the inhibitory activity further declined, with MGI values ranging from 45.81% (*E. coli*) to 72.45% (*S. aureus*). At the lowest tested concentration (62.5 µL/L), MGI values ranged from 38.45% (*C. krusei*) to 51.96% (*C. tropicalis*), confirming a concentration-dependent reduction in antimicrobial activity against all tested microorganisms.

### 3.5. Effect of LCCO on Microbiological Quality of White Radish During Refrigerated Storage

#### 3.5.1. Total Viable Count and Coliform Bacteria

**Figure 5:**
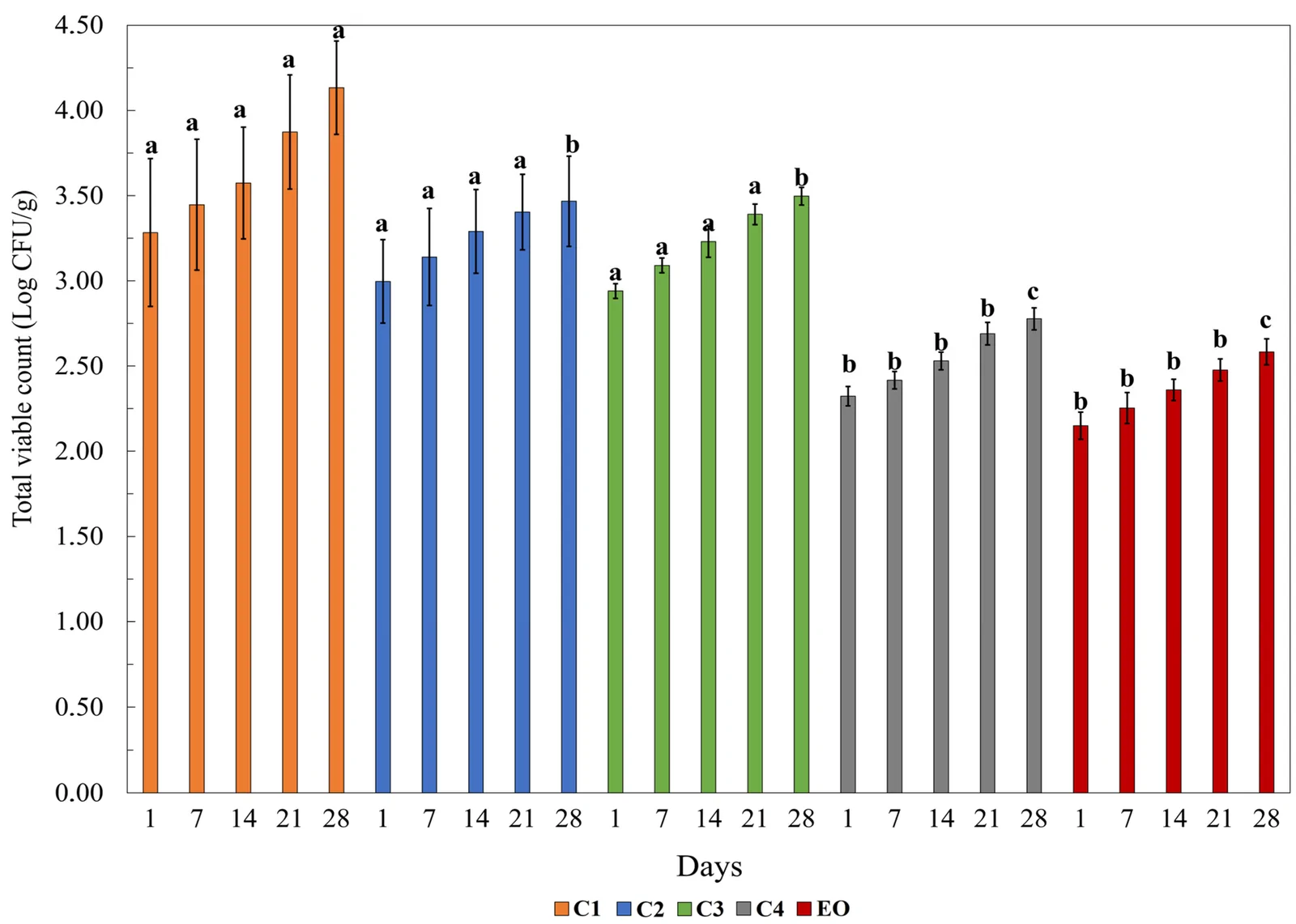
Total bacteria count (log CFU/g) in white radish samples measured on the days 1, 7, 14, 21, 28. Data represent the mean of three samples, with bars indicating the standard deviation. Different letters within each day indicate significant differences between the treatments (Tukey, *p* ≤ 0.05). C1 = non-vacuum packaged samples without LCCO or Tween 80 (−V/−LCCO/−Tween); C2 = vacuum-packaged samples without LCCO (+V/−LCCO/−Tween); C3 = non-vacuum packaged samples treated with Tween 80 solution only (−V/+Tween/−LCCO); C4 = non-vacuum packaged samples treated with LCCO (−V/+LCCO/+Tween); EO = vacuum-packaged samples treated with LCCO (+V/+LCCO/+Tween).

**Figure 6:**
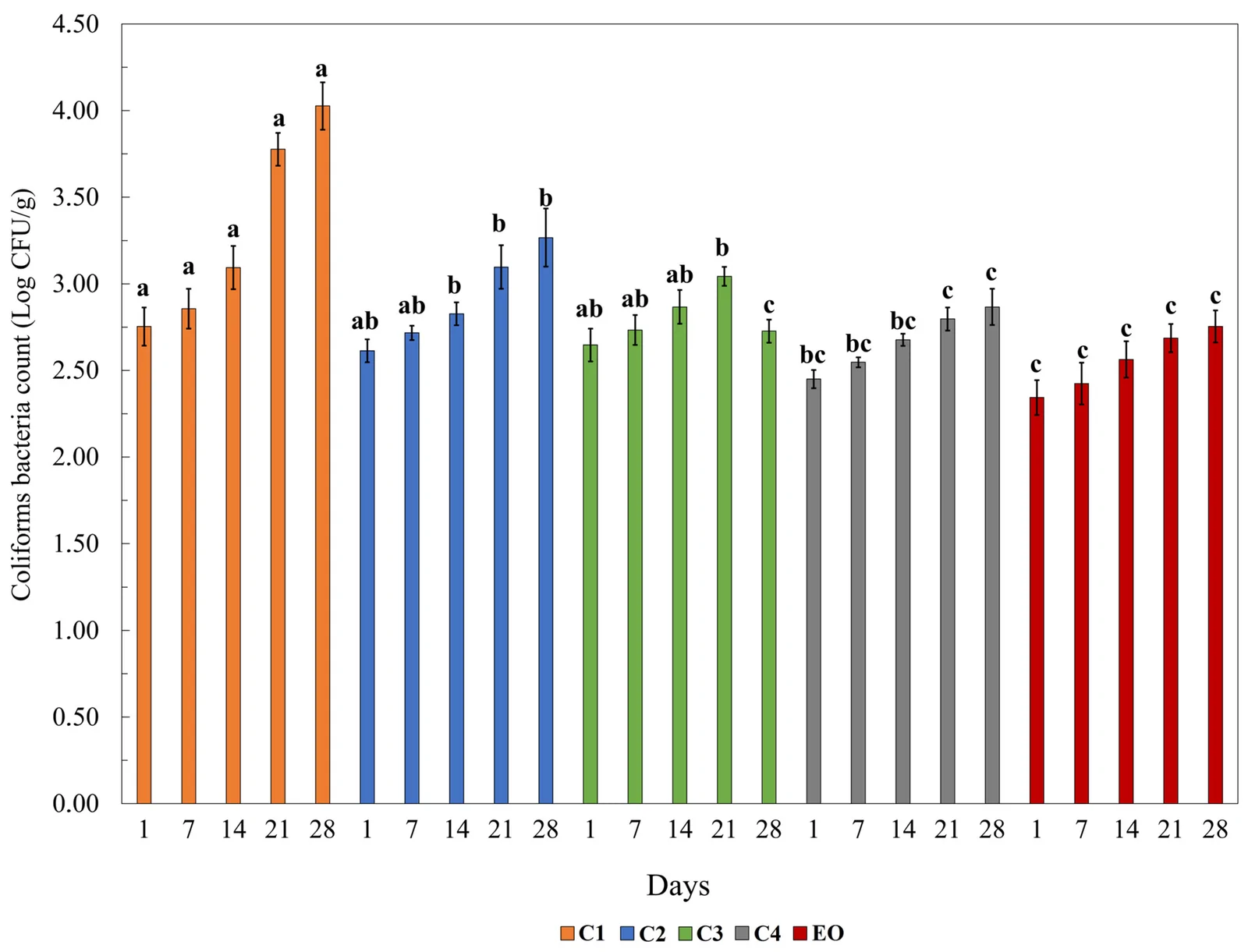
Coliform bacteria count (log CFU/g) in white radish samples measured on the days 1, 7, 14, 21, 28. Data represent the mean of three samples, with bars indicating the standard deviation. Different letters within each day indicate significant differences between the treatments (Tukey, *p* ≤ 0.05). C1 = non-vacuum packaged samples without LCCO or Tween 80 (−V/−LCCO/−Tween); C2 = vacuum-packaged samples without LCCO (+V/−LCCO/−Tween); C3 = non-vacuum packaged samples treated with Tween 80 solution only (−V/+Tween/−LCCO); C4 = non-vacuum packaged samples treated with LCCO (−V/+LCCO/+Tween); EO = vacuum-packaged samples treated with LCCO (+V/+LCCO/+Tween).

The effect of LCCO application and vacuum packaging on the microbiological quality of fresh white radish during 28 days of refrigerated storage at 4 °C is presented in Figure 5 and Figure 6. To further assess the effects of the treatments throughout storage, a two-way ANOVA was performed considering treatment and storage time as fixed factors, including their interaction (treatment × storage time). As reported in Table S1, both treatment and storage time significantly affected total viable bacterial counts (*p* < 0.0001), whereas their interaction was not significant (*p* = 0.978). This indicates that the overall microbial load increased significantly during storage, while the relative effect of the different treatments remained consistent over time. In contrast, for coliform counts, significant effects of both treatment and storage time were observed (*p* < 0.0001), together with a significant treatment × storage time interaction (*p* < 0.0001), indicating that the effectiveness of the treatments against coliform bacteria varied throughout storage.

Overall, the untreated control (C1) generally showed the highest microbial counts, particularly at the later stages of storage, whereas the LCCO-containing treatments (C4 and EO) maintained lower microbial loads throughout the experiment. Vacuum packaging alone (C2) provided an intermediate level of microbial control, while the effect of Tween 80 alone (C3) varied depending on the microbial parameter considered and the storage time. These findings suggest that LCCO application contributed to maintaining a lower microbial load during refrigerated storage, with its effect being particularly evident for total viable bacteria. The different response observed for coliform bacteria highlights that the efficacy of the treatments may depend on the target microbial group. The specific trends observed for total viable and coliform bacteria are discussed below in relation to Figure 5 and Figure 6.

On day 0, the baseline TVC and coliform bacteria counts of the untreated samples were 2.75 and 3.28 log CFU/g, respectively.

On day 1, TVC values ranged from 2.34 log CFU/g (EO) to 2.75 log CFU/g (C1), while coliform bacteria counts ranged from 2.15 log CFU/g (EO) to 3.28 log CFU/g (C1). Throughout the storage period, C1 and C3 exhibited the highest microbial counts, with TVC reaching 4.03 log CFU/g and coliform bacteria reaching 4.13 log CFU/g in C1 by day 28. The microbial counts in C3 were comparable to those in C1 at all sampling points, confirming that Tween 80, at the concentration used, exhibited no antimicrobial activity. Vacuum packaging alone (C2) resulted in a moderate reduction in both TVC and coliform bacteria counts compared with C1, with TVC and coliform bacteria counts of 3.27 and 3.47 log CFU/g, respectively, on day 28.

Application of LCCO without vacuum packaging (C4) resulted in consistently lower microbial counts than both control groups throughout storage, with TVC and coliform bacteria counts of 2.87 and 2.78 log CFU/g, respectively, on day 28. The combined application of LCCO and vacuum packaging (EO) resulted in the lowest microbial counts at all sampling time points, with TVC and coliform bacteria counts of 2.75 and 2.58 log CFU/g, respectively, on day 28, representing the most effective preservation treatment evaluated in the present study.

Microscopic fungi and yeasts were not detected in any experimental group throughout the storage period. Likewise, *Salmonella* spp. was not detected in any sample at any sampling point.

#### 3.5.2. Identification of Microorganisms by MALDI-TOF MS

**Figure 7:**
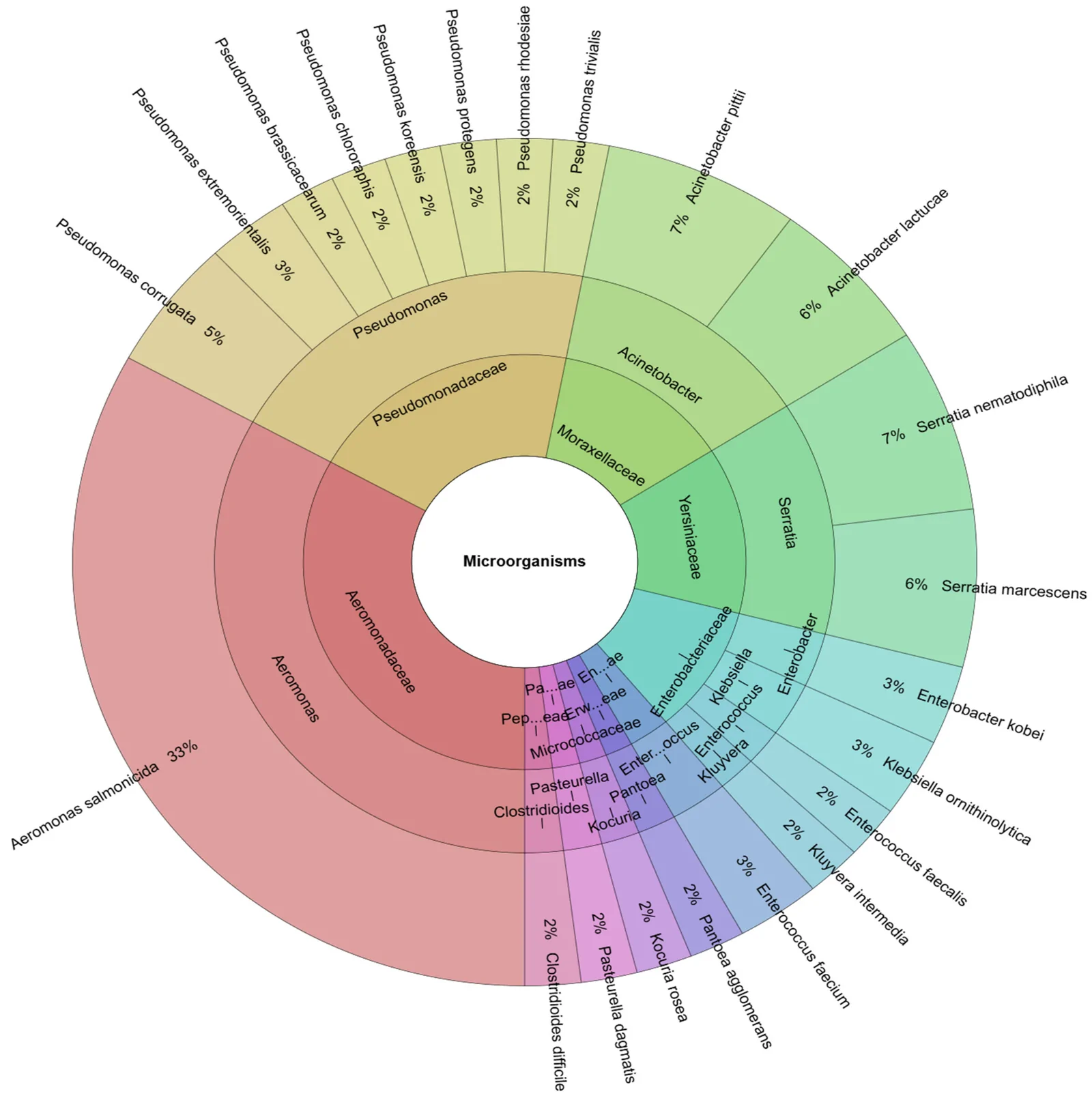
Taxonomic composition of bacterial isolates identified from fresh white radish by MALDI-TOF MS Biotyper at the family, genus, and species level.

A total of 404 bacterial isolates were identified at the species level using MALDI-TOF MS Biotyper (Figure 7). The identified microbiota comprised 22 species belonging to 12 genera and 10 families. The predominant species was *Acinetobacter pittii* (33%), followed by *Pantoea agglomerans* (7%), *Serratia nematodiphila* (7%), *Serratia marcescens* (6%), and *Acinetobacter lactucae* (6%).

At the genus level, *Pseudomonas* was the most species-rich genus, represented by eight species—*P. brassicacearum*, *P. corrugata*, *P. extremorientalis*, *P. chlororaphis*, *P. koreensis*, *P. protegens*, *P. rhodesiae*, and *P. trivialis*—collectively accounting for 19% of all identified isolates. At the family level, Moraxellaceae and Pseudomonadaceae were the predominant families, followed by Yersiniaceae and Enterobacteriaceae.

The remaining isolates were represented by *Aeromonas salmonicida* (3%), *Enterococcus faecium* (3%), *Enterococcus faecalis* (3%), *Clostridioides difficile* (2%), *Kluyvera intermedia* (2%), *Enterobacter kobei* (2%), *Kocuria rosea* (2%), *Pasteurella dagmatis* (2%), *Klebsiella ornithinolytica* (2%), and *Pseudomonas rhodesiae* (2%). The remaining species were each represented by fewer than seven isolates.

A total of 404 bacterial isolates were identified at the species level using MALDI-TOF MS Biotyper and were distributed among five experimental groups: Control (*n* = 141), Control vacuum (*n* = 65), Control Tween (*n* = 97), Control EO (*n* = 72), and EO vacuum (*n* = 29). The identified microbiota comprised 22 species belonging to 12 genera and 10 families (Table 4).

The predominant species across all groups was *Acinetobacter pittii* (33%), with the highest abundance in the Control group (45 isolates) and progressively lower numbers in the Control Tween (37 isolates), Control vacuum (26 isolates), Control EO (21 isolates), and EO vacuum (5 isolates) groups. *Pantoea agglomerans* (7%) was the second most abundant species, with the highest number of isolates in the Control group (12 isolates) and the lowest in the EO vacuum group (1 isolate). *Serratia nematodiphila* (7%) and *Serratia marcescens* (6%) were detected in all experimental groups, with both species represented by only three isolates in the EO vacuum group. *Acinetobacter lactucae* accounted for 6% and was relatively evenly distributed among the experimental groups.

Species of the genus *Pseudomonas* were detected exclusively or predominantly in the non-vacuum groups. No isolates of *P. brassicacearum*, *P. corrugata*, *P. extremorientalis*, *P. chlororaphis*, *P. koreensis*, *P. protegens*, or *P. rhodesiae* were recovered from the EO vacuum group. The remaining species, including *Aeromonas salmonicida*, *Clostridioides difficile*, *Enterococcus faecalis*, *Enterococcus faecium*, *Enterobacter kobei*, *Klebsiella ornithinolytica*, *Kluyvera intermedia*, *Kocuria rosea*, and *Pasteurella dagmatis*, were represented by low numbers of isolates (8–12 isolates per species) and were detected at lower frequencies in the treated groups than in the untreated control (Table 4).

### 3.6. Insecticidal Activity of LCCO

The insecticidal activity of LCCO against *C. maculatus* and *M. dorsalis* is presented in Table 5. LCCO exhibited concentration-dependent insecticidal activity against both tested species. Against *C. maculatus*, mortality increased from 7.33% at the lowest tested concentration (3.125%) to 94.33% at the highest concentration (100%). Similarly, mortality of *M. dorsalis* increased from 5.33% at 3.125% to 93.33% at 100%.

At intermediate concentrations, *C. maculatus* exhibited mortality rates of 77.00% at 50%, 58.67% at 25%, 35.00% at 12.5%, and 25.67% at 6.25%. *M. dorsalis* showed mortality rates of 79.00% at 50%, 51.00% at 25%, 27.67% at 12.5%, and 14.67% at 6.25%.

Probit analysis revealed an LC_50_ of 17.50% and an LC_90_ of 83.10% for *C. maculatus*, whereas the corresponding values for *M. dorsalis* were 20.99% and 87.60%, respectively. These results indicate that *C. maculatus* was slightly more susceptible to LCCO than *M. dorsalis*.

## 4. Discussion

### 4.1. Chemical Composition of LCCO

The present study demonstrated that *Ligusticum chuanxiong* CO_2_ oil (LCCO) possesses a phthalide-rich volatile profile dominated by cis-ligustilide (63.3%), followed by senkyunolide (15.8%), 3-butylidenephthalide (5.4%), and neocnidilide (4.9%). Together, these four phthalides accounted for more than 89% of the total chromatographic area, confirming that phthalides represent the major chemical constituents of LCCO. This composition is consistent with previous reports describing supercritical CO_2_ extracts of *L. chuanxiong*, in which cis-ligustilide is invariably the predominant constituent, although its relative abundance varies depending on geographical origin, harvest conditions, post-harvest processing, and extraction methodology [9].

Compared with hydrodistilled essential oils, the LCCO analysed in the present study contained only trace amounts of monoterpene hydrocarbons, reflecting the higher selectivity of supercritical CO_2_ extraction towards thermolabile lipophilic phthalides while reducing thermal degradation and isomerisation during extraction. Similar observations have been reported by Wang et al. [9], who demonstrated that CO_2_ extraction generally yields preparations enriched in ligustilide and other phthalides compared with conventionally distilled oils.

In addition to the major phthalides, beta-eudesmene (1.5%) represented the principal non-phthalide constituent, whereas all remaining terpenoids were detected only at low concentrations. The predominance of phthalides is particularly relevant because ligustilide, butylidenephthalide, and structurally related compounds have been recognised as the principal bioactive constituents responsible for the antimicrobial, antifungal, antioxidant, and insecticidal activities of *L. chuanxiong* extracts [10,22]. Therefore, the chemical profile identified in the present study provides a plausible explanation for the broad-spectrum antimicrobial, insecticidal, and food preservation activities demonstrated by LCCO in the subsequent experiments.

### 4.2. In Vitro Antimicrobial Activity

In the disc diffusion assay, the largest inhibition zones were observed for Gram-positive bacteria, with *E. faecalis* (14.33 mm), *S. aureus* (13.67 mm), and *L. monocytogenes* (13.33 mm) showing the greatest susceptibility. In contrast, Gram-negative bacteria exhibited smaller inhibition zones, ranging from 9.67 mm (*S. enterica*) to 11.67 mm (*E. coli*), while *Candida* spp. showed the lowest values (7.67–8.33 mm). The greater susceptibility of Gram-positive bacteria than Gram-negative species observed in the present study is consistent with the structural differences in bacterial cell envelopes. The outer membrane of Gram-negative bacteria, enriched in lipopolysaccharides, acts as an effective permeability barrier that restricts the diffusion of lipophilic compounds such as phthalides into the cell, thereby reducing their antimicrobial efficacy [23]. Similar susceptibility patterns have been reported for phthalide-rich and other lipophilic essential oils evaluated using the disc diffusion method [24,25,26,27].

The broth microdilution assay confirmed the antimicrobial activity observed in the disc diffusion test. *Y. enterocolitica* exhibited the lowest MIC_50_ value (0.064 mg/mL), whereas *C. tropicalis* was the least susceptible microorganism (MIC_50_ 3.492 mg/mL). The remarkably low MIC_50_ value obtained for *Y. enterocolitica* is noteworthy, given the generally higher tolerance of Gram-negative bacteria to lipophilic antimicrobials. This increased susceptibility may be associated with differences in the composition or permeability of the outer membrane compared with other Gram-negative species. Durofil et al. [23] similarly reported considerable variability in the susceptibility of *Y. enterocolitica* to plant-derived antimicrobials, indicating that certain essential oil constituents are capable of overcoming the permeability barrier of this foodborne pathogen.

The antimicrobial activity of LCCO is most likely associated with its phthalide-rich composition, particularly the high contents of cis-ligustilide and 3-butylidenephthalide. Mao et al. [28] demonstrated that butylidenephthalide disrupts microbial energy metabolism and promotes reactive oxygen species accumulation, providing a plausible mechanistic explanation for the broad-spectrum antimicrobial activity observed in the present study. Likewise, Wang et al. [22] identified ligustilide and structurally related phthalides as the principal antimicrobial constituents of *L. chuanxiong* essential oil, confirming that the biological activity resides predominantly within the phthalide fraction rather than the minor monoterpene components.

Considerable variability was also observed among *Candida* species, with MIC_50_ values ranging from 0.575 mg/mL for *C. glabrata* to 3.492 mg/mL for *C. tropicalis*. Such interspecies differences have been attributed to variations in cell wall composition, membrane ergosterol content, and the expression of efflux pump systems, which influence susceptibility to lipophilic antimicrobial compounds [29]. The antifungal activity of LCCO is further supported by Sim and Shin [30], who demonstrated additive to synergistic effects of ligustilide and butylidenephthalide in combination with azole antifungal agents against *Trichophyton* spp., and by Wang et al. [22], who confirmed the antifungal activity of phthalide-rich fractions from *L. chuanxiong* against *C. albicans*.

### 4.3. In Situ Vapour-Phase Antimicrobial Activity

The strong concentration-dependent vapour-phase antimicrobial activity of LCCO across all four tested food matrices confirms the suitability of phthalide-rich CO_2_ oils for *in situ* application as headspace antimicrobials in sealed produce packaging. The vapour-phase delivery approach is particularly well-suited to root vegetables and other fresh produce, where direct surface application of essential oils at bactericidal concentrations typically causes unacceptable organoleptic alterations; volatile release into the sealed headspace allows antimicrobial exposure of microbial cells on the food surface without direct contact between the oil and the food matrix [31]. Hoca et al. [31] synthesised evidence from 82 studies on vapour-phase essential oil applications in vegetable preservation and confirmed that this delivery mode effectively suppresses spoilage bacteria and moulds across diverse vegetable matrices, while identifying controlled release and concentration maintenance as the principal technical challenges for large-scale implementation. The methodology employed in the present study, including filter paper disc impregnation with serial dilutions of LCCO in ethyl acetate, hermetic sealing with Parafilm, and image analysis-based MGI quantification is consistent with the validated *in situ* methodology established for this research group and previously applied in evaluations of multiple essential oils on fresh vegetable matrices [24,25,26,27]. The consistently higher MGI values observed on white radish and carrot compared to pear and banana at equivalent headspace concentrations reflect the known influence of food matrix composition on vapour-phase antimicrobial efficacy: in fruit matrices with higher surface moisture and water activity, competitive absorption of volatile phthalide compounds by the food surface may reduce the effective concentration available for antimicrobial activity, whereas the lower water activity of root vegetable surfaces facilitates more effective microbial exposure to headspace volatiles. This matrix-dependent variability in *in situ* efficacy has been consistently observed for other essential oils evaluated by the same methodology across multiple food matrices including pear, banana, potato, kohlrabi, carrot, and celery [25,26]. The inhibitory activity of LCCO vapour against *L. monocytogenes* and *S. aureus* under *in situ* conditions is particularly relevant for fresh produce food safety given the documented association of these pathogens with fresh vegetable-related foodborne illness outbreaks and their capacity for growth under refrigerated storage temperatures.

### 4.4. Microbiological Quality of White Radish During Refrigerated Storage

The present study demonstrated that the combination of LCCO and vacuum packaging was the most effective treatment for preserving the microbiological quality of fresh white radish during 28 days of refrigerated storage. This combined treatment consistently resulted in lower total viable counts and coliform bacteria counts than either LCCO or vacuum packaging applied individually, indicating that both preservation strategies contributed to microbial inhibition.

The improved preservation efficacy can be explained by the complementary mechanisms of the two treatments. LCCO provides antimicrobial activity through the continuous release of volatile phthalide compounds into the package headspace, whereas vacuum packaging limits oxygen availability and suppresses the growth of aerobic spoilage microorganisms. Similar synergistic effects have been reported for other combinations of essential oils and oxygen-restrictive packaging systems. Yun et al. [32] demonstrated that microencapsulated essential oils combined with passive modified atmosphere packaging significantly reduced microbial growth and prolonged the shelf life of table grapes, highlighting the benefits of integrating natural antimicrobials with modified storage atmospheres.

The absence of any preservative effect in the Tween control confirmed that the reduction in microbial counts resulted from LCCO rather than from the emulsifying agent. This observation agrees with previous studies confirming that Tween 80 at concentrations used for essential oil emulsification does not exhibit intrinsic antimicrobial activity, functioning exclusively as an emulsifying agent [33]. Furthermore, *Salmonella* spp., microscopic fungi, and yeasts were not detected throughout storage in any treatment group. This finding is most likely associated with the low initial microbial contamination of the commercially obtained white radish and the hygienic handling of samples before storage.

Overall, these results demonstrate that combining LCCO with vacuum packaging provides greater microbiological stability than either preservation strategy alone and supports the potential application of this approach for extending the shelf life of minimally processed white radish.

### 4.5. Microbiota Composition of White Radish

Species-level identification of 404 bacterial isolates using MALDI-TOF MS Biotyper revealed a taxonomically diverse spoilage microbiota comprising 22 species belonging predominantly to Gram-negative bacteria associated with fresh vegetables. MALDI-TOF MS has become a reliable and rapid method for species-level identification of food-associated bacteria and has proven particularly useful for characterising the microbiota of fresh produce [34,35,36].

*Acinetobacter pittii* was the predominant species (33% of all isolates), followed by *Pantoea agglomerans*, *Serratia nematodiphila*, and *Serratia marcescens*. The predominance of these taxa agrees with previous reports describing plant-associated and psychrotrophic bacteria as major components of the microbiota of fresh vegetables during refrigerated storage [36,37]. The occurrence of *Acinetobacter* spp. is of particular interest because these bacteria are widely distributed in soil and on plant surfaces and have also been recognised as potential reservoirs of antimicrobial resistance in the food chain [38]. Likewise, *P. agglomerans* and *Serratia* spp. are common members of the epiphytic microbiota of vegetables and may contribute to spoilage during refrigerated storage [39,40].

One of the most notable findings of the present study was the distribution of *Pseudomonas* species among the experimental groups. Although eight *Pseudomonas* species were recovered from the untreated and partially treated samples, no *Pseudomonas* isolates were detected in the group treated with the combination of LCCO and vacuum packaging. Since *Pseudomonas* spp. are recognised as the principal spoilage bacteria of aerobically stored fresh vegetables owing to their psychrotrophic growth, aerobic metabolism, and production of extracellular degradative enzymes [41,42], their absence in the EO vacuum group suggests that this combined preservation strategy was particularly effective in limiting the recovery of this important spoilage genus.

In contrast, *Acinetobacter*, *Serratia*, and *Pantoea* species were still recovered from the EO vacuum group, although in markedly lower numbers than in the untreated control. This observation suggests that these taxa may exhibit greater tolerance to the combined treatment than *Pseudomonas* spp., although the mechanisms underlying this difference remain to be elucidated.

### 4.6. Insecticidal Activity

The present study demonstrated that LCCO exhibits concentration-dependent fumigant insecticidal activity against *Callosobruchus maculatus* and *Megabruchidius dorsalis*, with *C. maculatus* showing slightly greater susceptibility than *M. dorsalis*. These findings extend the documented biological activities of *Ligusticum chuanxiong* CO_2_ oil and indicate its potential for application in the protection of stored agricultural commodities.

The insecticidal activity observed in the present study is consistent with previous reports identifying ligustilide and 3-butylidenephthalide as the principal bioactive constituents responsible for the insecticidal properties of *L. chuanxiong*. Chu et al. [43] demonstrated that Z-ligustilide and Z-3-butylidenephthalide exhibited pronounced contact toxicity against *Sitophilus zeamais*, confirming that the phthalide fraction is primarily responsible for the pesticidal activity of *L. chuanxiong* essential oil. The fumigant activity observed in the present study is also consistent with the relatively high volatility of these compounds, enabling their diffusion through the package headspace and subsequent exposure of insects without direct contact.

The slightly higher LC_50_ and LC_90_ values obtained for *M. dorsalis* suggest lower susceptibility of this species to LCCO than *C. maculatus*. Although the underlying mechanisms were not investigated, species-specific differences in cuticle permeability, respiratory physiology, or detoxification capacity may contribute to the observed differences, as previously suggested for fumigant essential oils against stored-product beetles [44].

Comparison with previous studies indicates that fumigant toxicity varies considerably among essential oils because of differences in their chemical composition. Ebadollahi et al. [44] reported substantially lower LC_50_ values for thymol- and carvacrol-rich *Satureja* essential oils against *C. maculatus*, whereas Krzyżowski et al. [45] reported an LC_50_ of 15.69 µL/L air for *Rosmarinus officinalis* essential oil rich in 1,8-cineole. Such differences most likely reflect variations in the composition and physicochemical properties of volatile constituents rather than differences in biological activity alone.

To the best of our knowledge, this is the first study to evaluate the fumigant insecticidal activity of *Ligusticum chuanxiong* CO_2_ oil against *C. maculatus* and *M. dorsalis* using probit analysis. Together with its antimicrobial and food preservation activities, these findings further support the multifunctional potential of LCCO as a natural bioactive agent for postharvest applications.

### 4.7. Limitations and Novelty of the Study

The present study has several limitations that should be considered when interpreting the results. The storage experiment was performed under controlled laboratory conditions using a single batch of commercial LCCO, and therefore the results may not fully reflect the natural variability of CO_2_ extracts obtained from different geographical origins or harvest seasons, as essential oil composition is well documented to vary substantially with geographic origin, climate, and extraction conditions [46]. In addition, the *in situ* vapour-phase antimicrobial evaluation was performed using reference strains, whereas naturally contaminated fresh produce contains complex microbial communities that may respond differently to essential oil vapours. Furthermore, sensory quality attributes, including colour, texture, aroma, and consumer acceptability, were not evaluated and should be investigated in future studies to facilitate practical application of LCCO in postharvest preservation. The insecticidal evaluation was limited to adult mortality after 24 h of fumigation and did not include sublethal effects on reproductive parameters, oviposition rate, or F1 emergence, which are important endpoints for comprehensive assessment of pest management potential [47,48]. Furthermore, given the reported chemical instability of cis-ligustilide under heat, light, oxygen and pH changes [9], the chemical composition of LCCO was not re-analysed after emulsification, incorporation into the storage trial, or exposure to the microbiological testing conditions. Consequently, it cannot be fully excluded that the phthalide profile of the oil in direct contact with the tested microorganisms and food matrix differed from that of the freshly analysed oil. Future studies should include GC-MS re-analysis of LCCO composition following emulsification and storage to confirm the stability of its active constituents under the applied experimental conditions.

Despite these limitations, the present study provides several original contributions. The originality of this study lies in its integrated experimental design, combining four complementary evaluation levels: *in vitro* antimicrobial activity, vapour-phase antimicrobial efficacy, insecticidal activity, and postharvest storage performance within a single framework applied to *Ligusticum chuanxiong* CO_2_ oil, a phthalide-rich extract previously studied mainly in a pharmacological context. This integrated approach is complemented by species-level characterisation of the spoilage microbiota of fresh white radish using MALDI-TOF MS Biotyper, identifying *Acinetobacter pittii* as the predominant bacterial species. Moreover, no *Pseudomonas* species were recovered from samples treated with the combination of LCCO and vacuum packaging, highlighting the effectiveness of this preservation strategy against one of the most important spoilage genera associated with fresh vegetables. Collectively, these findings demonstrate the multifunctional potential of LCCO as a natural bioactive agent for postharvest preservation of fresh produce and provide a foundation for future studies focused on sensory evaluation, scale-up, and commercial application.

Unlike previous studies on LCCO and related *L. chuanxiong* extracts (Table S2), which focused mainly on the antifungal or pharmacological activity of the extract itself [8,10,12,13,22,28,30], the present study is the first to link the chemical composition of the extract with a species-level shift in the spoilage microbiota of the food matrix, as determined by MALDI-TOF MS. This provides new insight into the selective effect of LCCO on the spoilage community under realistic storage conditions.

## 5. Conclusions

The present study demonstrated that *Ligusticum chuanxiong* CO_2_ oil (LCCO), characterised by a phthalide-rich composition dominated by cis-ligustilide (63.3%), possesses significant antimicrobial, insecticidal, and food preservation potential. *In vitro* evaluation confirmed inhibitory activity against all tested bacterial and yeast reference strains, with *Y. enterocolitica* and *S. aureus* exhibiting the greatest susceptibility. *In situ* vapour-phase application at 500 µL/L achieved microbial growth inhibition exceeding 90% on white radish and carrot. Insecticidal fumigation assays demonstrated concentration-dependent mortality against *C. maculatus* (LC_50_ = 17.50%) and *M. dorsalis* (LC_50_ = 20.99%), highlighting the potential of LCCO as a natural grain protectant. The storage experiment demonstrated that the combined application of LCCO and vacuum packaging was the most effective treatment for reducing total viable counts and coliform bacteria on fresh white radish during 28 days of refrigerated storage at 4 °C. MALDI-TOF MS Biotyper analysis of 404 bacterial isolates revealed a diverse spoilage microbiota comprising 22 species, with *Acinetobacter pittii* as the predominant species. No *Pseudomonas* species were recovered from samples treated with the combination of LCCO and vacuum packaging. This study provides the first comprehensive evaluation of *Ligusticum chuanxiong* CO_2_ oil as a multifunctional postharvest agent by integrating antimicrobial, insecticidal, food preservation, and species-level microbiota analyses. Overall, these findings demonstrate the potential of LCCO, particularly in combination with vacuum packaging, as a multifunctional natural preservative for postharvest applications.

## Figures and Tables

**Table 1:** Chemical composition of LCCO as determined by GC-MS.

N.	Compound	LRI^calc^	LRI^lit^	Relative %
1	*α*-pinene	940	942	tr
2	*β*-pinene	968	970	tr
3	α-terpinene	1012	1015	0.1 ± 0.02
4	*p*-cymene	1030	1026	0.2 ± 0.02
5	limonene	1033	1031	0.2 ± 0.02
6	*γ*-terpinene	1060	1062	0.3 ± 0.02
7	terpinolene	1081	1084	0.1 ± 0.02
8	terpinene-4-ol	1179	1182	0.1 ± 0.02
9	2-methoxy4-vinylphenol	1280	1282	0.9 ± 0.04
10	*α*-cubebene	1349	1351	0.4 ± 0.03
11	*β*-elemene	1390	1393	0.1 ± 0.03
12	*α*-himachalene	1445	1447	0.1 ± 0.01
13	*β*-eudesmene	1479	1481	1.5 ± 0.07
14	*α*-selinene	1510	1512	0.3 ± 0.02
15	spathulenol	1600	1601	0.5 ± 0.03
16	3-butylphthalide	1630	1629	3.5 ± 0.10
17	3-butylidenephthalide	1681	1685	5.4 ± 0.15
18	senkyunolide	1721	1719	15.8 ± 1.01
19	*trans*-neocnidilide	1736	1735	4.9 ± 0.20
20	*cis*-ligustilide	1761	1758	63.3 ± 5.14
21	*trans*-ligustilide	1790	1788	2.2 ± 0.11
	**TOTAL**			**99.9**

The components are reported according to their elution order an apolar column; LRI^calc^: Linear Retention Indices measured an apolar column; LRI^lit^: Linear Retention indices from literature; Tr: percentage mean values ≤ 0.1%.

**Table 2:** Antimicrobial activity (expressed as inhibition zone in mm) of antibiotics (ATB) and *Ligusticum chuanxiong* CO_2_ oil (LCCO), evaluated by the disc diffusion method.

Class	Microorganism	ATB	LCCO
G-negative bacteria	*Escherichia coli* CCM 3954	30.33 ± 0.58 ^bcd,A^	11.67 ± 0.59 ^bc,B^
	*Salmonella enterica* CCM 3807	32.67 ± 0.59 ^a,A^	9.67 ± 0.58 ^de,B^
	*Yersinia enterocolitica* CCM 7204^T^	30.67 ± 0.57 ^bc,A^	10.33 ± 0.58 ^cd,B^
G-positive bacteria	*Enterococcus faecalis* CCM 4224	31.67 ± 0.58 ^ab,A^	14.33 ± 0.58 ^a,B^
	*Listeria monocytogenes* CCM 4699	30.33 ± 0.59 ^bcd,A^	13.33 ± 0.58 ^ab,B^
	*Staphylococcus aureus* CCM 4223	31.67 ± 0.57 ^ab,A^	13.67 ± 0.57 ^a,B^
Yeast	*Candida albicans* CCM 8186	29.33 ± 0.58 ^cde,A^	7.67 ± 0.59 ^f,B^
	*Candida glabrata* CCM 8270	28.67 ± 0.59 ^de,A^	8.33 ± 0.59 ^ef,B^
	*Candida krusei* CCM 8271	29.67 ± 0.57 ^cde,A^	8.33 ± 0.58 ^ef,B^
	*Candida parapsilosis* CCM 8260	30.67 ± 0.58 ^bc,A^	7.67 ± 0.57 ^f,B^
	*Candida tropicalis* CCM 8223	28.33 ± 0.58 ^e,A^	8.33 ± 0.58 ^ef,B^

Data are the mean (±standard deviation) of 3 replicates. ^a–f^ Different superscript lowercase letters indicate statistically significant differences within each column (*p* ≤ 0.05). ^A,B^ Different superscript uppercase letters indicate statistically significant differences within each row (*p* ≤ 0.05).

**Table 3:** Minimum inhibitory concentrations (MIC_50_ and MIC_90_) of LCCO, expressed in mg/mL.

Class	Microorganism	MIC_50_	MIC_90_
G-negative bacteria	*Escherichia coli*	1.848 ± 0.091 ^d^	1.977 ± 0.117 ^cd^
	*Salmonella enterica*	2.270 ± 0.094 ^c^	2.773 ± 0.280 ^b^
	*Yersinia enterocolitica*	0.064 ± 0.016 ^g^	0.151 ± 0.015 ^f^
G-positive bacteria	*Enterococcus faecalis*	1.342 ± 0.075 ^e^	1.803 ± 0.149 ^cd^
	*Listeria monocytogenes*	1.763 ± 0.118 ^d^	2.000 ± 0.069 ^c^
	*Staphylococcus aureus*	0.288 ± 0.063 ^g^	1.198 ± 0.049 ^e^
Yeast	*Candida albicans*	2.558 ± 0.185 ^b^	2.896 ± 0.052 ^b^
	*Candida glabrata*	0.575 ± 0.110 ^f^	0.929 ± 0.067 ^e^
	*Candida krusei*	2.732 ± 0.049 ^b^	2.970 ± 0.016 ^b^
	*Candida parapsilosis*	1.258 ± 0.062 ^e^	1.649 ± 0.084 ^d^
	*Candida tropicalis*	3.492 ± 0.078 ^a^	3.800 ± 0.056 ^a^

Data are the mean (±standard deviation) of 3 replicates. ^a–g^ Different superscript lowercase letters indicate statistically different values within the column (*p* ≤ 0.05). LCCO = *Ligusticum chuanxiong* CO_2_ oil.

**Table 4:** Distribution of bacterial species identified by MALDI-TOF MS Biotyper from fresh white radish across experimental groups.

Microorganisms	Control	Control Vacuum	Control Tween	Control EO	EO Vacuum	Number
*Acinetobacter lactucae*	6	5	6	4	2	23
*Acinetobacter pittii*	45	26	37	21	5	134
*Aeromonas salmonicida*	4	2	2	2	2	12
*Clostridioides difficile*	2	2	2	2	2	10
*Enterobacter kobei*	2	2	2	1	1	8
*Enterococcus faecalis*	3	2	2	2	2	11
*Enterococcus faecium*	4	2	2	2	2	12
*Klebsiella ornithinolytica*	2	2	2	2	2	10
*Kluyvera intermedia*	2	2	1	2	1	8
*Kocuria rosea*	2	2	1	2	1	8
*Pantoea agglomerans*	12	5	7	3	1	28
*Pasteurella dagmatis*	3	2	2	1	1	9
*Pseudomonas brassicacearum*	4	0	2	2	0	8
*Pseudomonas corrugata*	10	0	5	5	0	20
*Pseudomonas extremorientalis*	6	0	3	3	0	12
*Pseudomonas chlororaphis*	4	0	2	2	0	8
*Pseudomonas koreensis*	5	0	1	2	0	8
*Pseudomonas protegens*	3	0	2	2	0	7
*Pseudomonas rhodesiae*	4	0	2	2	0	8
*Pseudomonas trivialis*	2	1	2	1	1	7
*Serratia marcescens*	7	5	6	4	3	25
*Serratia nematodiphila*	9	5	6	5	3	28
**Total**	**141**	**65**	**97**	**72**	**29**	**404**

**Table 5:** Mortality (expressed in %) at different concentrations of LCCO against *Callosobruchus maculatus* and *Megabruchidius dorsalis*.

Concentration of LCCO (%)	*C. maculatus*	*M. dorsalis*
3.125	7.33 ± 0.58 ^f,A^	5.33 ± 1.15 ^f,B^
6.25	25.67 ± 4.04 ^e,A^	14.67 ± 2.08 ^e,B^
12.5	35.00 ± 8.19 ^d,A^	27.67 ± 4.04 ^d,A^
25	58.67 ± 3.59 ^c,A^	51.00 ± 3.61 ^c,B^
50	77.00 ± 4.58 ^b,A^	79.00 ± 2.65 ^b,A^
100	94.33 ± 5.63 ^a,A^	93.33 ± 5.51 ^a,A^

Data represent the mean (± standard deviation) of 3 replicates. ^a–f^ Different superscript lowercase letters indicate statistically different values within the column (*p* ≤ 0.05). ^A,B^ Different superscript uppercase letters indicate statistically different values within the row (*p* ≤ 0.05). LCCO = *Ligusticum chuanxiong* CO_2_ oil.

## Data Availability

The original contributions presented in this study are included in the article/Supplementary Material. Further inquiries can be directed to the corresponding authors.

## Supplementary Materials

The following supporting information can be downloaded at: https://www.mdpi.com/article/10.3390/foods15162936/s1, Table S1: Two-way ANOVA results for the effects of treatment, storage time, and their interaction on total viable bacterial and coliform counts during storage; Table S2: Comparison of the present study with previous studies on *Ligusticum chuanxiong* extracts and essential oil.

## References

[1] Bianchi A., Venturi F. (2025). Enhancing the Shelf Life of Food Products: Strategies, Challenges, and Innovations. Foods.

[2] Kumar A., Solanki M.K., Santoyo G. (2025). Editorial: Beneficial Microbes for Sustainable Postharvest Management of Fresh Produce. Front. Microbiol..

[3] Abbas A., Guo Y., Ji N., Hao S., Xu H., Asim M. (2026). Natural Antimicrobials as Sustainable Preservatives against Postharvest Microbial Spoilage in Fruits and Vegetables. Food Biosci..

[4] Bianchi A., Sanmartin C., Taglieri I., Macaluso M., Venturi F., Napoli M., Mancini M., Fabbri C., Zinnai A. (2023). Effect of Fertilization Regime of Common Wheat (*Triticum aestivum*) on Flour Quality and Shelf-Life of PDO Tuscan Bread. Foods.

[5] Innova Market Insights (2025). Global Clean Label Trends. https://www.innovamarketinsights.com/trends/global-clean-label-trends/.

[6] Rabbani A., Khaliq A., Mudgil P., Maqsood S., Nazir A. (2026). Recent Advances in Lemongrass Essential Oil: Food Safety, Preservation, and Bioactivity in Food Systems. Comp. Rev. Food Sci. Food Safe.

[7] Kačániová M., Galovičová L., Schwarzová M., Čmiková N. (2023). Antimicrobial Effects of Rosemary Essential Oil with Potential Use in the Preservation of Fresh Fruits and Vegetables. Acta Hortic. Regiotect..

[8] Chen Z., Zhang C., Gao F., Fu Q., Fu C., He Y., Zhang J. (2018). A Systematic Review on the Rhizome of *Ligusticum chuanxiong* Hort. (Chuanxiong). Food Chem. Toxicol..

[9] Wang Y., Wu L., Wang H., Jiang M., Chen Y., Zheng X., Li L., Yin Q., Han L., Bai L. (2025). *Ligusticum chuanxiong*: A Chemical, Pharmacological and Clinical Review. Front. Pharmacol..

[10] Cui K., He Y., Wang M., Li M., Jiang C., Wang M., He L., Zhang F., Zhou L. (2023). Antifungal Activity of *Ligusticum chuanxiong* Essential Oil and Its Active Composition Butylidenephthalide against *Sclerotium rolfsii*. Pest Manag. Sci..

[11] Zuo J., Zhang T.-H., Peng C., Xu B., Dai O., Lu Y., Zhou Q.-M., Xiong L. (2024). Essential Oil from *Ligusticum chuanxiong* Hort. Alleviates Lipopolysaccharide-Induced Neuroinflammation: Integrating Network Pharmacology and Molecular Mechanism Evaluation. J. Ethnopharmacol..

[12] Kong Q., Niu Y., Feng H., Yu X., Wang B., Liu X., Chen Y., Wang F., Tian J., Zhou H. (2024). *Ligusticum chuanxiong* Hort.: A Review of Its Phytochemistry, Pharmacology, and Toxicology. J. Pharm. Pharmacol..

[13] Chen H., Zhao Y., Qin G., Bi Y., Yue G., Zhang M., Chang X., Qiu X., Luo L., Yang C. (2022). Antifungal Effects and Active Components of *Ligusticum chuanxiong*. Molecules.

[14] Monacci E., Sanmartin C., Bianchi A., Pettinelli S., Najar B., Mencarelli F., Taglieri I. (2025). Chemical Quality and Characterization of Essential Oils in Postharvest Hop Cv. Cascade: Ventilated Room Temperature as a Sustainable Alternative to Hot-Stove and Freeze-Drying Processes. Beverages.

[15] Kačániová M., Vukovic N.L., Čmiková N., Galovičová L., Schwarzová M., Šimora V., Kowalczewski P.Ł., Kluz M.I., Puchalski C., Bakay L. (2023). *Salvia sclarea* Essential Oil Chemical Composition and Biological Activities. Int. J. Mol. Sci..

[16] Kačániová M., Čmiková N., Vukovic N.L., Verešová A., Bianchi A., Garzoli S., Ben Saad R., Ben Hsouna A., Ban Z., Vukic M.D. (2024). *Citrus limon* Essential Oil: Chemical Composition and Selected Biological Properties Focusing on the Antimicrobial (*In Vitro*, *In Situ*), Antibiofilm, Insecticidal Activity and Preservative Effect against *Salmonella enterica* Inoculated in Carrot. Plants.

[17] Kačániová M., Galovičová L., Valková V., Ďuranová H., Borotová P., Štefániková J., Vukovic N.L., Vukic M., Kunová S., Felsöciová S. (2021). Chemical Composition and Biological Activity of *Salvia officinalis* Essential Oil. Acta Hortic. Regiotect..

[18] (2022). Microbiology of the Food Chain: Horizontal Method for the Enumeration of Microorganisms—Part 1: Colony Count at 30 °C by the Pour Plate Technique.

[19] (2006). Microbiology of Food and Animal Feeding Stuffs—Horizontal Method for the Enumeration of Coliforms—Colony-Count Technique.

[20] (2008). Microbiology of Food and Animal Feeding Stuffs—Horizontal Method for the Enumeration of Yeasts and Moulds.

[21] (2017). Microbiology of the Food Chain—Horizontal Method for the Detection, Enumeration and Serotyping of Salmonella.

[22] Wang Z., Tang X., Lv L., Qiao S., Chen M., Song H. (2025). HPTLC-Bioautography–MS-Guided Strategy for the Detection of Phthalides with Antimicrobial and Antioxidant Activities From *Ligusticum chuanxiong* Essential Oil. Phytochem. Anal..

[23] Durofil A., Maddela N.R., Naranjo R.A., Radice M. (2022). Evidence on Antimicrobial Activity of Essential Oils and Herbal Extracts against *Yersinia enterocolitica*—A Review. Food Biosci..

[24] Kačániová M., Vukovic N., Ban Z., Li L., Ben Hsouna A., Ben Saad R., Elizondo-Luevano J.H., Bianchi A., Popovic S., Garzoli S. (2025). *Lavandula angustifolia* Essential Oil: GC—MS Composition, *In Vitro* Antimicrobial Activity, Vapor-Phase Efficacy on Fruit and Vegetable Models, and Anti-*Escherichia coli* Effect in Sous-Vide Potatoes. J. Food Saf..

[25] Kačániová M., Galovičová L., Borotová P., Vukovic N.L., Vukic M., Kunová S., Hanus P., Bakay L., Zagrobelna E., Kluz M. (2022). Assessment of *Ocimum basilicum* Essential Oil Anti-Insect Activity and Antimicrobial Protection in Fruit and Vegetable Quality. Plants.

[26] Kačániová M., Galovičová L., Valková V., Ďuranová H., Štefániková J., Čmiková N., Vukic M., Vukovic N.L., Kowalczewski P.Ł. (2022). Chemical Composition, Antioxidant, *In Vitro* and *In Situ* Antimicrobial, Antibiofilm, and Anti-Insect Activity of *Cedar atlantica* Essential Oil. Plants.

[27] Verešová A., Terentjeva M., Ban Z., Li L., Vukic M., Vukovic N., Kluz M.I., Ben Sad R., Ben Hsouna A., Bianchi A. (2024). Enhancing Antimicrobial Efficacy of Sandalwood Essential Oil Against *Salmonella enterica* for Food Preservation. Foods.

[28] Mao X., Chen L., Wang M., Cao T., Cui K., Zhang G., Cao L., Zhang J., Zhou L. (2025). Identification of the Isocitrate Lyase SrICL as a Potential Target Protein of Butylidenephthalide in *Sclerotium rolfsii* via Drug Affinity Responsive Target Stability and Metabolomics. J. Agric. Food Chem..

[29] Karpiński T.M., Ożarowski M., Seremak-Mrozikiewicz A., Wolski H. (2023). Anti-*Candida* and Antibiofilm Activity of Selected Lamiaceae Essential Oils. Front. Biosci..

[30] Sim Y., Shin S. (2008). Combinatorial Anti-Trichophyton Effects of *Ligusticum chuanxiong* Essential Oil Components with Antibiotics. Arch. Pharm. Res..

[31] Hoca G., Bozik M., Mascellani Bergo A., Bozikova K., Kloucek P. (2026). Vapour-Phase Essential Oils for Postharvest Vegetables: From Laboratory to Real-World Use. LWT.

[32] Yun X., Zhang L., Dong T., Zhang H., Sun T., Hu J., Cheng P. (2025). Configuration Of A Combined Microencapsulated Essential Oil Antimicrobials with Modified Atmosphere Biodegradable Films for Shelf-Life Extension of Table Grapes. Food Sci. Nutr..

[33] Nielsen C.K., Kjems J., Mygind T., Snabe T., Meyer R.L. (2016). Effects of Tween 80 on Growth and Biofilm Formation in Laboratory Media. Front. Microbiol..

[34] El-Nemr I.M., Mushtaha M., Sundararaju S., Fontejon C., Suleiman M., Tang P., Goktepe I., Hasan M.R. (2019). Application of MALDI Biotyper System for Rapid Identification of Bacteria Isolated from a Fresh Produce Market. Curr. Microbiol..

[35] Singhal N., Kumar M., Kanaujia P.K., Virdi J.S. (2015). MALDI-TOF Mass Spectrometry: An Emerging Technology for Microbial Identification and Diagnosis. Front. Microbiol..

[36] Surányi B.B., Taczman-Brückner A., Mohácsi-Farkas C., Engelhardt T. (2023). Rapid Identification of Bacteria from Agricultural Environment Using MALDI-TOF MS. Acta Aliment..

[37] Touchon M., Cury J., Yoon E.-J., Krizova L., Cerqueira G.C., Murphy C., Feldgarden M., Wortman J., Clermont D., Lambert T. (2014). The Genomic Diversification of the Whole *Acinetobacter* Genus: Origins, Mechanisms, and Consequences. Genome Biol. Evol..

[38] Ababneh Q., Al-Rousan E., Jaradat Z. (2022). Fresh Produce as a Potential Vehicle for Transmission of *Acinetobacter baumannii*. Int. J. Food Contam..

[39] Gillis A., Rodríguez M., Santana M.A. (2014). Serratia Marcescens Associated with Bell Pepper (*Capsicum annuum* L.) Soft-Rot Disease under Greenhouse Conditions. Eur. J. Plant Pathol..

[40] Shikov A.E., Merkushova A.V., Savina I.A., Nizhnikov A.A., Antonets K.S. (2023). The Man, the Plant, and the Insect: Shooting Host Specificity Determinants in *Serratia marcescens* Pangenome. Front. Microbiol..

[41] Barth M., Hankinson T.R., Zhuang H., Breidt F., Sperber W.H., Doyle M.P. (2009). Microbiological Spoilage of Fruits and Vegetables. Compendium of the Microbiological Spoilage of Foods and Beverages.

[42] Raposo A., Pérez E., De Faria C.T., Ferrús M.A., Carrascosa C., Singh O.V. (2016). Food Spoilage by *Pseudomonas* spp.—An Overview. Foodborne Pathogens and Antibiotic Resistance.

[43] Chu S.S., Jiang G.H., Liu Z.L. (2011). Insecticidal Components from the Essential Oil of Chinese Medicinal Herb, *Ligusticum chuanxiong* Hort. J. Chem..

[44] Ebadollahi A., Naseri B., Aghcheli A., Setzer W.N. (2025). Insecticidal Efficacy of *Satureja hortensis* L. and *Satureja khuzistanica* Jamzad Essential Oils Against *Callosobruchus maculatus* (F.). Plants.

[45] Krzyżowski M., Baran B., Łozowski B., Francikowski J. (2020). The Effect of *Rosmarinus officinalis* Essential Oil Fumigation on Biochemical, Behavioral, and Physiological Parameters of *Callosobruchus maculatus*. Insects.

[46] Abdelmohsen U.R., Elmaidomy A.H. (2025). Exploring the Therapeutic Potential of Essential Oils: A Review of Composition and Influencing Factors. Front. Nat. Prod..

[47] Ferraz M.S.S., Faroni L.R.D., De Sousa A.H., Heleno F.F., Silva M.V.D.A., De Alencar E.R. (2024). Toxicity of *Piper hispidinervum* Essential Oil to *Callosobruchus maculatus* and Cowpea Bean Quality. Plants.

[48] Viteri Jumbo L.O., Haddi K., Faroni L.R.D., Heleno F.F., Pinto F.G., Oliveira E.E. (2018). Toxicity to, Oviposition and Population Growth Impairments of *Callosobruchus maculatus* Exposed to Clove and Cinnamon Essential Oils. PLoS ONE.

