# Supplementary material for: Ligusticum chuanxiong CO2 Oil as a Natural Antimicrobial Agent for Postharvest Preservation of Fresh White Radish: In Vitro, In Situ and Storage Evaluation
Source: Foods. 2026 Aug 21;15(16):2936. doi: 10.3390/foods15162936 (PMC13511980; doi:10.3390/foods15162936)
Supplement: Supplementary file 1 [file foods-15-02936-s001.zip › foods-4489285-supplementary.pdf]

Table S1. Two-way ANOVA results for the effects of treatment, storage time, and their interaction on total viable bacterial and coliform counts during storage.

Factors	Total viable count F	*p*-value	Significance	Coliform count F	p-value	Significance
Treatment	107.59	<0.0001	***	135.57	<0.0001	***
Storage time	18.20	<0.0001	***	104.16	<0.0001	***
Treatment × Storage time	0.40	0.978	ns	13.47	<0.0001	***

Two-way ANOVA was performed with treatment and storage time as fixed factors. Significant effects were considered at $p \leq 0.05$.

Table S2. Comparison of the present study with previous studies on *Ligusticum chuanxiong* extracts and essential oil.

Study	Extract / EO type	Levels evaluated
Chen et al. 2018 [8]	*L. chuanxiong* rhizome (review)	Systematic review of chemical constituents and toxicology, no original antimicrobial or postharvest data
Cui et al. 2023 [10]	LCCO / butylidenephthalide	In vitro antifungal activity (*Sclerotium rolfsii*)
Kong et al. 2024 [12]	*L. chuanxiong* (review)	Review of phytochemistry, pharmacology and toxicology, no original antimicrobial or postharvest data
Chen et al. 2022 [13]	*L. chuanxiong* extract	*In vitro* antifungal effects and active component identification
Wang et al. 2025 [22]	*L. chuanxiong* essential oil	In vitro antimicrobial and antioxidant activity (HPTLC bioautography)
Mao et al. 2025 [28]	Butylidenephthalide	Mechanistic study (target protein identification), in vitro
Sim and Shin 2008 [30]	*L. chuanxiong* essential oil	*In vitro* anti-Trichophyton activity, combination with antibiotics
**Present study**	**LCCO**	***In vitro* antimicrobial activity, vapour-phase antimicrobial efficacy, insecticidal activity, 28-day postharvest storage trial, MALDI-TOF MS microbiota identification**
